# Variation in the quality and out-of-pocket cost of treatment for childhood malaria, diarrhoea, and pneumonia: Community and facility based care in rural Uganda

**DOI:** 10.1371/journal.pone.0200543

**Published:** 2018-11-26

**Authors:** Seyi Soremekun, Frida Kasteng, Raghu Lingam, Anna Vassall, Edmound Kertho, Stella Settumba, Patrick L. Etou, Agnes Nanyonjo, Guus ten Asbroek, Karin Kallander, Betty Kirkwood, on behalf of the inSCALE Study Group

**Affiliations:** 1 Department of Population Health, London School of Hygiene and Tropical Medicine, London United Kingdom; 2 Department of Global Health and Development, London School of Hygiene and Tropical Medicine, London United Kingdom; 3 Division of Global Health, Department of Public Health Sciences, Karolinska Institutet, Stockholm, Sweden; 4 Institute of Health and Society, Newcastle University, Newcastle, United Kingdom; 5 The Malaria Consortium, Kampala, Uganda; 6 National Perinatal Epidemiology and Statistics Unit, University of New South Wales, Sidney, Australia; 7 African Population Health Research Centre, Nairobi, Kenya; 8 Department of Global Health, Academic Medical Centre, Amsterdam, The Netherlands; 9 The Malaria Consortium, London, United Kingdom; Instituto Rene Rachou, BRAZIL

## Abstract

**Background:**

A key barrier to appropriate treatment for malaria, diarrhoea, and pneumonia (MDP) in children under 5 years of age in low income rural settings is the lack of access to quality health care. The WHO and UNICEF have therefore called for the scale-up of integrated community case management (iCCM) using community health workers (CHWs). The current study assessed access to treatment, out-of-pocket expenditure and the quality of treatment provided in the public and private sectors compared to national guidelines, using data collected in a large representative survey of caregivers of children in 205 villages with iCCM-trained CHWs in mid-Western Uganda.

**Results:**

The prevalence of suspected malaria, diarrhoea and suspected pneumonia in the preceding two weeks in 6501 children in the study sample were 45%, 11% and 24% respectively. Twenty percent of children were first taken to a CHW, 56% to a health facility, 14% to other providers and no care was sought for 11%. The CHW was more likely to provide appropriate treatment compared to any other provider or to those not seeking care for children with MDP (RR 1.51, 95% CI 1.42–1.61, p<0.001). Seeking care from a CHW had the lowest cost outlay (median $0.00, IQR $0.00-$1.80), whilst seeking care to a private doctor or clinic the highest (median $2.80, IQR $1.20-$6.00). We modelled the expected increase in overall treatment coverage if children currently treated in the private sector or not seeking care were taken to the CHW instead. In this scenario, coverage of appropriate treatment for MDP could increase in total from the current rate of 47% up to 64%.

**Conclusion:**

Scale-up of iCCM-trained CHW programmes is key to the provision of affordable, high quality treatment for sick children, and can thus significantly contribute to closing the gap in coverage of appropriate treatment.

## Introduction

Together pneumonia, diarrhoea and malaria account for nearly a third of all deaths in children below the age of five years [1, 2], the majority of which occur in sub-Saharan Africa [3–5]. Prompt and correct treatment of these conditions is the most effective way to reduce mortality [6]. Treatment should be delivered at high coverage and quality if the most at-risk populations are to achieve successful control of these diseases–universal health coverage being a key aim of the Sustainable Development Goals [7]. However, ensuring adequate access to treatment still proves challenging in low and middle income settings particularly as existing health facility-based services alone have not been able to address the coverage gap in appropriate treatment. There is therefore an urgent need for financially accessible, expanded health provision “close to home” that will facilitate equitable coverage of poorer, rural communities [8].

The World Health Organisation and UNICEF have championed the integrated management of common childhood illnesses in the community (iCCM) [6, 9]. Through the iCCM strategy, community health workers (CHWs) are trained and provided with the tools to diagnose and treat cases of childhood pneumonia, malaria and diarrhoea as a complement to existing facility-based health systems [10]. Since 2014 at least 28 countries in sub-Saharan Africa have incorporated iCCM into their national health policies and many have implemented regional iCCM programmes [11]. The projected benefits of such programmes could be significant. However, there is as yet little robust evidence evaluating the relative usage, performance, and particularly the cost benefit to the user, of community health workers trained in iCCM in comparison to existing facility-based or other care that is locally available. Such evidence will prove essential in understanding the barriers and facilitators to appropriate treatment of children with in the health system as a whole.

The current study was part of the inSCALE project, an integrated 6.5-years research programme which aimed to improve the appropriate treatment of children under 5 years of age for suspected malaria, diarrhoea and suspected pneumonia (MDP) through additional, innovative supportive supervision-based interventions for CHWs already trained and active in iCCM (inSCALE Clinical Trials Identifier: NCT01810055 http://clinicaltrials.gov/show/NCT01810055). The inSCALE programme was implemented in 8 districts in mid-Western Uganda where Uganda’s iCCM programme was operational. Uganda operates a decentralised model of health care, with services operating at the national and subnational level and provided by both private and government-run facilities [12]. At the lowest level of care are the Village Health Team members (VHT), a voluntary cadre of CHW which are located in communities with otherwise poor access to health facilities [13]. In its 2010–2015 Health Sector Strategic and Investment Plan (HSSIP), Uganda’s Ministry of Health (MoH) renewed their commitment to maintaining the VHT programme as a key player in both health promotion and in improving sickness prevention awareness and treatment coverage in the maternal and child health space [14]. Historically however such voluntary health staff models have suffered from poor supportive supervision from facility or health unit staff, and difficulties in retaining and motivating staff [15–17]—and Uganda is no different [14]. The HSSIP proposed to address this by focusing on the improvement of the supervision, resources and training of VHTs, with better integration of the service within the wider health system and linkage to the community [14]. Given the significant presence of private health services of varying quality in the same rural areas in which VHTs operate, and the general household trend towards private care-seeking [18, 19], a key indicator of success of the VHT programme will be its impact on sector-wide health indicators in this changing environment.

In the iCCM areas in the eight study site districts, one to two VHTs per community had been trained to manage simple childhood illnesses and to identify and refer children with malnutrition. It is in this context that the current study aims to explore the associations between care-seeking location, out-of-pocket treatment costs, and management of childhood illness. We report on the coverage of appropriate treatment for children with MDP, the out-of-pocket costs to caretakers of sick children, and the relative performance and contribution of the region’s iCCM-VHT programme under these indicators in comparison with other existing health providers.

## Methods

### Study setting and design

A description of the study context and site is provided elsewhere [20]. In brief, iCCM implementation has been supported in 8 districts in mid-Western Uganda by the Malaria Consortium (a leading not for profit international organization specialising in maternal and child health) through a grant from the Canadian International Development Agency (CIDA) since August 2010. Following this and prior to the roll-out of the inSCALE interventions, a cross sectional survey was undertaken across the 8 participating districts. To be eligible for the inSCALE study, a sub-county had to have VHTs trained in iCCM by 31st January 2011. We excluded sub-counties that contained less than 10 villages and those where other Malaria Consortium projects had operated to avoid respondent fatigue. This yielded an overall total of 41 eligible sub-counties active in iCCM for surveillance.

Five villages, with an additional five back-up villages were randomly selected per sub-county for surveillance. A list of all households in each community was supplied by the local parish council and verified by the field supervisor. From this list thirty-two households per community were selected and surveyed at random between May and August 2011 in accordance with the sample size requirements for the evaluation of the inSCALE trial [20].

### Household surveys

The analyses presented in this paper are based on data collected in this 2011 inSCALE cross-sectional survey. Data on socioeconomic and demographic characteristics of households, symptoms of the most recent illness episode in children under 5 years of age in the two weeks preceding the survey, care seeking behaviour, treatments received and details of all the self-reported out-of-pocket costs associated with care seeking for the episode of illness, were collected from the primary carer. The survey questionnaires were based on Demographic Health Survey (DHS)/Multiple Indicator Cluster Survey (MICS) child health and heath economics survey instruments used extensively in low and middle income countries [21, 22], and are provided in S1 Questionnaire File. Draft versions of the questionnaire were piloted on site and updated in an iterative process to ensure intended meanings were accurately conveyed. Pictures of locally available drugs for common childhood illnesses were used to increase the accuracy of recall of treatments received for sick children (S1 Fig). The questionnaires were delivered in the local languages of the region (Luganda, Luo and Runyakitara).

To ensure consistency, 10% of household interviews were repeated by field supervisors within a week of the original and discrepancies resolved. All questionnaires were double entered into a dedicated database and differences between copies verified and corrected. Range and consistency checks were run on data to ensure that missing/incorrect fields were identified and flagged for resolution.

Case definitions of episodes of suspected malaria, diarrhoea and suspected pneumonia, appropriate treatment for each of these diagnoses, care seeking and cost of care seeking were developed in accordance with standard WHO/UNICEF guidelines for treatment of malaria, diarrhoea and pneumonia in the community and at health facilities [10, 23–26]. Detailed definitions are provided in S1A–S1D Table. In brief, cases of suspected malaria episodes include all children with reported fever, excluding those with a negative malaria blood-test, suspected pneumonia episodes include all children with reported fast breathing and cough, and those with an episode of diarrhoea will have passed at least three watery stools in a 24hr period during the illness episode. The survey allowed for interviewees to record all the care seeking locations visited for the illness episode in question, and follow-on questions on transport and costs incurred whilst seeking care were asked for the first two locations visited. Definitions of out-of-pocket cost are provided in S1D Table.

### Data analysis

Analyses were based on caregiver-reported episodes of suspected malaria, diarrhoea or suspected pneumonia (this was the most recent episode in the two weeks prior to the interview). The difference in proportions of appropriate treatment between care seeking locations (by combining all episodes of MDP, and again separated by type of illness episode) was evaluated using logistic regression, with the VHT as the treatment group (or ‘exposure of interest’), and other care providers as the null group. Regression models were calculated using general estimating equations with an exchangeable correlation structure to account for sub-county clusters. Relative risks were derived from the regression models by use of the marginal standardisation technique, and the 95% CIs estimated with the delta method [27]. There are a range of demand-side characteristics that could influence the chances of appropriate treatment for sick children, including household income, parents’ education, rural versus urban location, age of parents and children, gender and others. We made the *a priori* decision not to force those of the above indicators for which we had data (mothers age, household income, education, religion, occupation, age and gender of child) into the final regression model as there is good evidence to show that these primarily operate via their influence on care seeking behaviour [28, 29]. The objective of this paper is not to determine the root causes of appropriate treatment, rather to ascertain how ultimate care seeking location influences the probability of appropriate treatment, and the implications for future scale-up of community case management programmes for childhood illnesses. We nonetheless provide a comparison of the demographic profiles of households by the care seeking location first visited for reference.

In accordance with the age-specific guidelines for classification and treatment of children (S1A and S1B Table) the samples for analysis was restricted to children between the ages of 2 months and 59 months, or 4 months and 59 months in the case of those with suspected or confirmed malaria. These represent the age ranges for community-based treatment of children with MDP.

After dropping data outliers, the household financial costs associated with seeking care were converted from Ugandan shillings to US dollars (using 2011 currency exchange rates [30]). Both median and mean out-of-pocket costs of care seeking were calculated to provide an indication of the spread of the data.

We assessed the theoretical overall improvement in appropriate treatment rates that could be gained from diverting those sick children for whom no care was sought or who were taken for care to the poorest-performing providers, to instead seeking care to the VHT as the first port of call. For this exercise, the overall appropriate treatment rate observed was described mathematically as the sum of the products of the proportions of MDP episodes taken to each care provider type (first port of call), and the proportions of episodes appropriately treated at the provider type in question (Eq 1). In each scenario, the children not seeking care or taken to poorly performing providers (i.e. β_i_ for each of such providers as below) were instead switched in turn to care seeking to the VHT, and the overall rate of appropriate treatment re-estimated.

$$Pc=\sum_{i}^{n}\beta_{i}*\gamma_{i}$$ Eq 1

*Pc*- Predicted coverage of appropriate treatment

*β*- Proportion of sick children (i.e. with fever, diarrhoea, or pneumonia) taken to provider *i*

*ϒ*- Proportion of children appropriately treated at provider *i*

n- Total number of provider types (including ‘no care seeking’) in study area

As a reference for future evaluations of iCCM programmes we calculated intra-class correlation coefficients (ICCs) to describe the distribution of the variation amongst children within and between sub-counties with respect to their probability of receiving appropriate treatment. These were calculated using the unweighted analysis of variance estimator (‘loneway’ command) in Stata 13.1: loneway calculates an ICC as a function of the F-statistic from a one way analysis of variance of appropriate treatment rate with cluster ID as the only predictor [31, 32].

Data were summarised as tables or Microsoft Excel (Microsoft Corp 2010) graphs, and statistical analysis carried out using Stata version 13.1 (StataCorp Texas USA).

### Ethics

Ethical approval was provided by the Higher Degrees, Research and Ethics Committee Uganda (IRB0011353: 2011), the Uganda National Council of Science and Technology (HS958: 2011), and the Ethics Committee of the London School of Hygiene and Tropical Medicine (ref 5762: 2010, 2011). Informed written consent was obtained from all participants.

## Results

### Prevalence of illness

A total of 3900 households in the study sample contained children under 5 years of age, representing 60% of 6553 households surveyed. These provided a total sample of 6501 children between the ages of 2 and 59 months (with a mean of 1.7 children per household), and 6262 children between the ages of 4 months and 59 months (the sub-sample in which suspected malaria symptoms and treatment would be evaluated). In total, 3347 children were reported as having symptoms of one or more of MDP in the preceding two weeks, with a total of 5057 episodes of MDP identified. The overall prevalence of sick children with symptoms of MDP in the preceding two weeks was 51%, and a breakdown by type of illness episode, and the total is shown in Table 1. A blood test for malaria (RDT or blood slide) was performed in 25% (741 children) of cases of fever with a rate of 86% (636 children) positivity (Table 1).

**Table 1:** Prevalence of children with fever, suspected malaria, diarrhoea or suspected pneumonia (MDP) and overall rates of appropriate testing (blood test for fever) and treatment. See S1C Table for additional definitions of appropriate treatment.

Type of illness	% prevalence (n)	% appropriately assessed or treated (n)
Children with fever*	47% (2921)	25%† (741)
Children with suspected malaria*^^^	45% (2816)	47% (1316)
Children with confirmed malaria*	86% (636)	73% (462)
Children with diarrhoea	11% (685)	30% (206) (ORS)
		9% (60) (ORS and zinc)
Children with pneumonia	24% (1556)	54% (840)
Episodes of MDP in total	5057	47% (2362)

*Denominator excludes children below 4months of age (same restriction applies in subsequent analyses).

^ Excludes 105 children who had a blood test which was reported as negative.

†This is the percentage of children with fever who received a blood test for malaria

Demographic characteristics are presented in Table 2. Characteristics of caregivers remained broadly similar when grouped according to the first location used to seek care for a sick child. Caretakers of sick children were mostly between 20 and 40 years of age, had some primary education or no formal education, were Christian, farmers or other manual labourers, and lived in households with less than 20USD income per month.

**Table 2:** Socio-demographic characteristics of caretakers of children with suspected malaria, diarrhoea, or suspected pneumonia; overall, and by first care seeking location.

	Overall*	VHT	Public facility	Private facility	Pharmacy	Other	No care sought outside home
Total caretakers	2102	409	529	666	173	127	198
**Age—caretaker**							
12–19	5% (110)	4% (17)	6% (34)	4% (29)	7% (12)	5% (6)	6% (12)
20–29	37% (772)	38% (155)	35% (185)	38% (255)	42% (73)	33% (42)	31% (62)
30–39	27% (570)	28% (113)	27% (142)	30% (197)	20% (34)	29% (37)	24% (47)
40–49	14% (295)	15% (61)	15% (80)	12% (83)	15% (26)	11% (14)	16% (31)
50+	12% (245)	12% (48)	10% (55)	11% (72)	13% (22)	19% (24)	12% (24)
age not possible to ascertain	5% (110)	4% (15)	6% (33)	5% (30)	3% (6)	3% (4)	11% (22)
**Highest educational level completed**								
No education	26% (555)	26% (106)	24% (126)	27% (183)	25% (43)	27% (34)	32% (63)
Some primary	49% (1029)	48% (195)	51% (269)	50% (331)	42% (73)	51% (65)	48% (96)
Completed primary	21% (431)	22% (92)	21% (111)	19% (125)	26% (45)	18% (23)	18% (35)
Secondary or above	4% (87)	4% (16)	4% (23)	4% (27)	7% (12)	4% (5)	2% (4)
**Religion**							
Christian	92% (1935)	94% (386)	92% (486)	92% (613)	90% (156)	88% (112)	92% (182)
Muslim	6% (135)	4% (17)	7% (37)	6% (42)	7% (12)	10% (13)	7% (14)
Traditional/other	2% (32)	1% (6)	1% (6)	2% (11)	3% (5)	2% (2)	1% (2)
**Occupation**								
Farmer/manual	88% (1858)	91% (372)	88% (466)	87% (578)	87% (151)	92% (117)	88% (174)
other	12% (244)	9% (37)	12% (63)	13% (88)	13% (22)	8% (10)	12% (24)
**Household monthly cash income**							
< USD 20	65% (1108)	69% (238)	70% (301)	61% (327)	54% (74)	67% (68)	65% (100)
USD 20–80	26% (440)	25% (88)	20% (87)	28% (150)	32% (44)	25% (26)	29% (45)
> USD 80	9% (153)	6% (21)	10% (41)	11% (56)	13% (18)	8% (8)	6% (9)

*Data derived from 2102 care seekers of 3336 sick children (excluded: 11 sick children where caretaker did not have full demographic data).

### Care seeking

Table 3 shows the first care provider visited during the most recent illness episode in children overall and by illness type. Care seeking patterns specifically for children with a fever irrespective of later suspected/confirmed malaria diagnosis can be found in S2 Table, and shows the same pattern of care seeking location preferences as those in Table 3.

**Table 3:** First care seeking location for children with suspected malaria, diarrhoea or suspected pneumonia.

Location	Overall Children (n = 3347)	Overall Episodes* (n = 5057)	Suspected malaria (n = 2816)	Diarrhoea (n = 685)	Suspected pneumonia (n = 1556)
	% (n)	% (n)	% (n)	% (n)	% (n)
VHT	20% (662)	19% (980)	19% (532)	19% (128)	21% (320)
Public facility	24% (803)	24% (1229)	25% (705)	24% (165)	23% (359)
Private facility/doctor	32% (1060)	33% (1687)	33% (916)	35% (241)	34% (530)
Pharmacy	8% (255)	7% (364)	8% (212)	6% (38)	7% (114)
General shop/other	6% (203)	6% (301)	6% (168)	6% (41)	6% (92)
No care sought	11% (364)	10% (496)	10% (283)	11% (72)	9% (141)

* Because a proportion of children in our sample exhibited symptoms of more than one condition, we have included the care seeking breakdown by both children and episodes for reference.

The majority of children with MDP were initially taken to a health facility or doctor (56%), which, in most cases was a privately-run clinic or doctor (32%). In total, 20% of respondents sought care from the VHT in the first instance; those who did not cited a perceived lack of drugs as the main reason (57%), followed by the VHT not being available (10%); these responses (both in type of reason and order of popularity) held even when stratified by reported symptoms of MDP. For 11% of children, no care was sought outside the home. The main reasons for no care seeking was that the family had no money (21%), the illness was perceived to be manageable at home with leftover or existing drugs (26%), or that the illness was not perceived to be severe (16%). These also remained the top three reasons when stratified by reported symptoms of MDP, though children with suspected malaria cited the use of existing drugs as the top reason for not seeking care, whilst those with diarrhoea or pneumonia cited a lack of money as the top reason.

Although the survey asked about all care seeking locations visited, the majority of sick children (73% 2442/3347) were taken to only one care seeking location, and the maximum number of care seeking visits reported for any episode of illness was two (16% 541/3347). Caretakers who first visited the VHT were most likely to go to a second provider for further advice or treatment (43% of the time): the most likely second care seeking locations after visiting the VHT were health facilities (19% and 17% of those who first visited a VHT went on to a private or public facility respectively). 21% of those who first went to a public facility went on to seek care elsewhere, compared to only 7% of those who first went to a private facility and 2% of those who first went to a pharmacy (Table 4).

**Table 4:** Second care seeking choice of children with suspected malaria, diarrhoea or suspected pneumonia, by first provider visited.

2^nd^ Location	Any 2^nd^ location	VHT	Public facility	Private facility or doctor	pharmacy	General shop/other	No second location
1^st^ Location (N)	% (n)	% (n)	% (n)	% (n)	% (n)	% (n)	% (n)
VHT (662)	43% (284)	0% (1)	17% (113)	19% (128)	4% (28)	2% (14)	57% (378)
Public facility (803)	21% (165)	2% (16)	2% (13)	13% (108)	2% (19)	1% (9)	79% (638)
Private facility or doctor (1060)	7% (73)	2% (23)	3% (36)	1% (7)	0% (1)	1% (6)	93% (987)
Pharmacy (255)	2% (6)	1% (3)	1% (2)	0% (1)	0% (0)	0% (0)	98% (249)
General shop/other (203)	6% (13)	2% (4)	2% (4)	1% (2)	0% (0)	1% (3)	94% (190)
No care sought (364)	-	-	-	-	-	-	-

### Appropriate treatment

There were 5057 cases of suspected malaria, diarrhoea or suspected pneumonia reported in total (Table 1). 47% (1316) of these cases were treated correctly–leaving a gap of 53% of cases treated inappropriately or not at all (Table 1). Numbers and percentages of children appropriately treated overall and by provider are presented in Tables 1 and 5 respectively. As Table 5 illustrates, of the 980 cases of MDP seen first by the VHT, 65% were appropriately treated. This is in comparison to only 42% of sick children taken elsewhere (out of 4077 sick children not taken to a VHT: RR 1.51, 95% CI 1.42–1.61, p<0.001). Of the 1229 children taken to a public facility 62% were appropriately treated (RR versus VHT 1.04, 95% CI 0.98–1.01, p = 0.238), 39% (of 1687 children) who were taken to a private facility were appropriately treated (RR versus VHT 1.62. 95% CI 1.47–1.78, p>0.001), 37% (of 364 children) were appropriately treated at a pharmacy (RR versus VHT 1.75, 95% CI 1.50–2.05, p<0.001), 36% (of 301 children) were appropriately treated at a general shop/other location (RR versus VHT 1.80, 95% CI 1.53–2.12, p<0.001) and 12% (of 496 children) who did not seek care outside the home (RR versus VHT 5.00, 95% CI 3.78–6.61, p < .0.001).

**Table 5:** Appropriate treatment of children with episodes of MDP by first provider where care was sought. Significance tests compare appropriate treatment rate at the VHT (‘exposure group’) versus other provider types (‘null group’) in turn.

First provider at which care sought	Total episodes treated at this provider	% episodes appropriately treated (n)	RR of VHT versus alternative provider(s) (95% CI)	P value
VHT	980	65% (635)		
Non-VHT (all below combined)	4077	42% (1727)	1.51 (1.42–1.61)	<0.001
Public health facility	1229	62% (764)	1.04 (0.98–1.10)	0.238
Private health facility/doctor	1687	39% (661)	1.62 (1.47–1.78)	<0.001
Pharmacy	364	37% (134)	1.75 (1.50–2.05)	<0.001
General shop/other	301	36% (107)	1.80 (1.53–2.12)	<0.001
No care sought	496	12% (61)	5.00 (3.78–6.61)	<0.001

Tables 6–8 present a breakdown of the percentages of sick children who received appropriate testing for malaria, and received appropriate treatment by illness type at each provider.

**Table 6:** Blood test performance rates and appropriate treatment of children with suspected or confirmed malaria by first provider where care was sought. Significance tests compare appropriate treatment rate at the VHT (‘exposure group’) versus other provider types (‘null group’) in turn.

First provider at which care sought	Total episodes treated at this provider	% episodes appropriately treated (n)	RR of VHT versus alternative provider(s) (95% CI)	P value
Blood test performed for cases of fever*				
VHT	591	53% (316)		
Non-VHT (all below combined)	2330	19% (425)	2.92 (2.57–3.32)	<0.001
Public health facility	735	36% (267)	1.46 (1.23–1.73)	<0.001
Private health facility/doctor	926	14% (128)	3.79 (3.06–4.70)	<0.001
Pharmacy	212	2% (5)	21.60 (8.88–52.56)	<0.001
General shop/other	174	14% (24)	3.94 (2.45–6.33)	<0.001
No care sought	283	0% (1)	134.70 (24.30–746.75)	<0.001
**Suspected malaria**				
VHT	532	70% (370)		
Non-VHT (all below combined)	2284	41% (946)	1.65 (1.52–1.80)	<0.001
Public health facility	705	68% (477)	1.02 (0.95–1.10)	0.517
Private health facility/doctor	916	33% (304)	2.04 (1.76–2.37)	<0.001
Pharmacy	212	34% (73)	2.03 (1.61–2.55)	<0.001
General shop/other	168	30% (51)	2.21 (1.69–2.89)	<0.001
No care sought	283	14% (41)	4.58 (3.41–6.15)	<0.001
**Confirmed malaria** ^				
VHT	257	86% (221)		
Non-VHT (all below combined)	379	64% (241)	1.34 (1.23–1.47)	<0.001
Public health facility	237	71% (169)	1.20 (1.11–1.29)	<0.001
Private health facility/doctor	118	48% (57)	1.75 (1.41–2.17)	<0.001
Pharmacy	5	60% (3)	1.44 (0.75–2.78)	0.278
General shop/other	18	67% (12)	1.22 (0.89–1.67)	0.222
No care sought	1	0% (0)	**n/a**	**-**

*2921 children with fever includes those who had a negative test (these are excluded from the definition of suspected malaria).

^ Numbers insufficient to conduct a comparison of the % appropriately treated at the VHT versus % appropriate treatment of those not seeking care.

**Table 7:** Appropriate treatment of children with diarrhoea with ORS or ORS plus zinc by first provider where care was sought. Significance tests compare appropriate treatment rate at the VHT (‘exposure group’) versus other provider types (‘null group’) in turn.

First provider at which care sought	Total episodes treated at this provider	% episodes appropriately treated (n)	RR of VHT versus alternative provider(s) (95% CI)	P value
**Diarrhoea treatment with ORS**				
VHT	128	54% (69)		
Non-VHT (all below combined)	557	25% (137)	2.17 (1.68–2.80)	<0.001
Public health facility	165	41% (67)	1.31 (1.02–1.67)	0.034
Private health facility/doctor	241	23% (56)	2.27 (1.60–3.23)	<0.001
Pharmacy	38	11% (4)	4.81 (2.19–10.55)	<0.001
General shop/other	41	17% (7)	3.49 (1.41–8.68)	0.007
No care sought	72	4% (3)	12.36 (3.56–42.94)	<0.001
**Diarrhoea treatment with ORS and zinc**				
VHT	128	24% (31)		
Non-VHT (all below combined)	557	5% (29)	4.71 (2.67–8.30)	<0.001
Public health facility	165	13% (21)	1.91 (1.08–3.37)	0.026
Private health facility/doctor	241	2% (6)	10.59 (4.31–26.02)	<0.001
Pharmacy	38	3% (1)	9.85 (1.18–82.11)	0.034
General shop/other	41	2% (1)	8.79 (1.55–50.01)	0.014
No care sought	72	0% (0)	**n/a**	**-**

**Table 8:** Appropriate treatment of children with suspected pneumonia by first provider where care was sought. Significance tests compare appropriate treatment rate at the VHT (‘exposure group’) versus other provider types (‘null group’) in turn.

First provider at which care sought	Total episodes treated at this provider	% episodes appropriately treated (n)	RR of VHT versus alternative provider(s) (95% CI)	P value
VHT	320	61% (196)		
Non-VHT (all below combined)	1236	52% (644)	1.18 (1.06–1.31)	0.003
Public health facility	359	61% (220)	1.00 (0.88–1.13)	0.991
Private health facility/doctor	530	57% (301)	1.08 (0.95–1.23)	0.240
Pharmacy	114	50% (57)	1.22 (0.99–1.49)	0.062
General shop/other	92	53% (49)	1.16 (0.95–1.41)	0.149
No care sought	141	12% (17)	4.98 (3.08–8.06)	<0.001

Across all illness categories, the VHT offered the highest level of appropriate treatment (24% to 86% of sick children received the correct treatment according to classification) compared to public health facilities (13% to 71%) and to the private sector (i.e. those seeking care to a private facility, a pharmacy or herbalist: 2% to 60%). In the majority of cases, these differences in appropriate treatment rates between VHTs and other providers were highly significant. As Table 2 additionally indicates, coverage of appropriate treatment varied highly by illness classification–for instance, 70% of children with diarrhoea did not receive ORS and more than 90% did not receive ORS plus zinc–the largest gaps in coverage of all treatment types.

The treatment outcomes of the 53% (2695) of cases of MDP that did not receive appropriate drugs are reported in S3 Table, and suggest that the majority (83% of 2237) of these received some form of treatment not recommended in the guidelines for the condition in question.

Overall, only 26% of children with fever received a diagnostic blood test (RDT or microscopy–Table 6). Those with fever who were first taken to the VHT were significantly more likely to have received a diagnostic blood test (53%) than at any other provider or compared to those not seeking care (blood test coverage at other providers/no care ranged from 0% - 36%, all p values <0.001 in comparison to the VHT). One caretaker of a child with fever who did not seek care reported that a blood test was carried out (Table 6)–this was not possible to verify further other than to confirm that the test was not an RDT.

Intra-cluster correlation coefficients (ICCs) for appropriate treatment can be found in S4 Table and ranged from 0.0167 to 0.0459. The appropriate treatment of suspected and confirmed malaria showed the most clustering (ICCs of 0.0328 and 0. 0459 respectively), whilst the lowest ICCs were observed for the appropriate treatment of diarrhoea with ORS and zinc (0.0203) and pneumonia (0.0167).

### Cost of care seeking

The overall out-of-pocket cost of seeking care for sick children (i.e. including the cost of both first and in some cases a second location) are presented in Table 9. The public facilities that children were taken to are disaggregated by primary (level II or III) or secondary (level IV or hospital) care levels as costs were accrued differently between the two (disaggregation was not possible for private sector care seeking as data was not collected by care level). Cost data were right-skewed with a few caretakers who sought care at higher level facilities reporting very high costs. Only 14% of caretakers who first sought care to a VHT reported expenditure in relation to the VHT visit, compared to 46% and 70% respectively of those that sought care at a public primary or secondary care facility and 100% of those that sought care at a private facility. Expenditure in relation to care seeking with a VHT was predominantly subsistence costs (occurred by 13% of households) and cost of medicines (2% of households). Subsistence, transportation and medicines were the most prevalent care seeking expenditures for public facilities (paid by 26%, 14% and 19% of households respectively in relation to a visit to a lower-level public facility and 42%, 37% and 33% respectively visiting higher-level facilities). At private facilities medicine costs was the most commonly occurring cost (93%), followed by subsistence costs (32%) and transport costs (15%). Those who first visited the VHT for the illness episode had the lowest overall costs associated with that illness episode (median (IQR) 0 (0–1.8), mean (SD) 2.0 (5.6)). Care seeking at a private facility resulted in the highest median cost outlays overall, USD 2.8 (IQR: 1.0–6.0). See S5 Table for a breakdown of mean costs by type of expenditure–this demonstrates that the cost of medicines was the largest outlay for families (means ranging from USD 1.24 to USD 3.59 by location), compared to costs for miscellaneous consumables/gratuities (means ranging from USD 0.00 to USD 0.29 by location). The highest overall mean costs of care seeking were reported from public secondary care facilities, at USD 5.7 (SD: 13.7). Five percent of caretakers seeking care to public secondary care facilities and 2% at private facilities reported total expenditures exceeding USD 25. The cost of seeking care to the first provider visited are shown in S6 Table. Seeking care from a VHT had the lowest cost outlay (median (IQR) 0 (0–0), mean (SD) USD 0.3 (1.2)) in comparison to all other provider types followed by lower level public sector facilities (median (IQR) USD 0 (0–1.2), mean (SD) USD 1.7 (USD 5.6)).

**Table 9:** Medical, non-medical and total out-of-pocket costs of seeking care for children with an episode of MDP, USD 2011 stratified by the first location visited.

First care seeking location	N^a^	Out-of-pocket costs in relation to seeking care for most recent illness episode					
		Median (IQR*)			Mean (SD*)		
		Medical **	Non-medical***	Total expenses	Medical	Non-medical	Total expenses
VHT (public sector level I)	n = 662	0.0 (0.0–0.6)	0.0 (0.0–0.2)	0.0 (0.0–1.8)	1.3 (4.4)	0.7 (2.1)	2.0 (5.6)
Public health facility, primary care only (public sector level II or III)	n = 655	0.0 (0.0–1.4)	0.0 (0.0–0.8)	0.2 (0.0–2.4)	2.0 (7.9)	0.9 (2.7)	2.9 (10.2)
Public health facility, with inpatient care (public sector level IV or hospital)	n = 143	0.0 (0.0–3.4)	0.4 (0.0–2.0)	1.4 (0.0–4.9)	3.1 (9.1)	2.6 (6.9)	5.7 (13.7)
Private health facility (clinic or hospital) or doctor	n = 1,059	2.3 (0.8–4.8)	0.0 (0.0–1.1)	2.8 (1.2–6.0)	3.9 (5.4)	1.3 (4.4)	5.2 (8.4)
Private pharmacy	n = 255	1.0 (0.2–2.4)	0.0 (0.0–0.0)	1.0 (0.4–3.0)	1.8 (2.5)	0.3 (1.1)	2.1 (2.9)
General shop/other	n = 202	1.4 (0.2–3.7)	0.0 (0.0–0.7)	1.9 (0.2–4.5)	2.8 (4.2)	0.7 (1.7)	3.6 (5.2)

^a^7 records dropped due to abnormally high costs (4 records) or missing cost data (3 records)

*IQR interquartile range, SD standard deviation

** registration fees, medicines, consumables, 'gratuities'

***transport, subsistence costs. Note public facilities are disaggregated by primary (level II or III) or secondary (level IV or hospital) care levels as costs were accrued differently between the two.

### The impact of increasing iCCM VHT care seeking on the coverage of appropriate treatment for suspected malaria, diarrhoea and suspected pneumonia in children under 5 years of age

In order to understand the extent to which increasing VHT care seeking could close the overall gap in the coverage of appropriate treatment for MDP, we re-calculated the percentage of children that would be appropriately treated for each of the three conditions if those children seeking care outside the public health system instead visited the VHT as their first port of call (all else being equal). We did not consider the effects of switching those who first sought care at public facilities to seeking care to the VHT, in order to concentrate on the poorest-performing providers (the VHT programme could also be seen as part of the wider public health system).

We thus did this for the four non-public provider care seeking options: i) private facilities/doctors, ii) pharmacies, iii) traditional healers/other providers, and iv) those not seeking care outside the home. We estimated the effect on appropriate treatment when children were switched to the VHT from each of these alternative options separately, and when children from all four were switched together.

**Fig 1:**
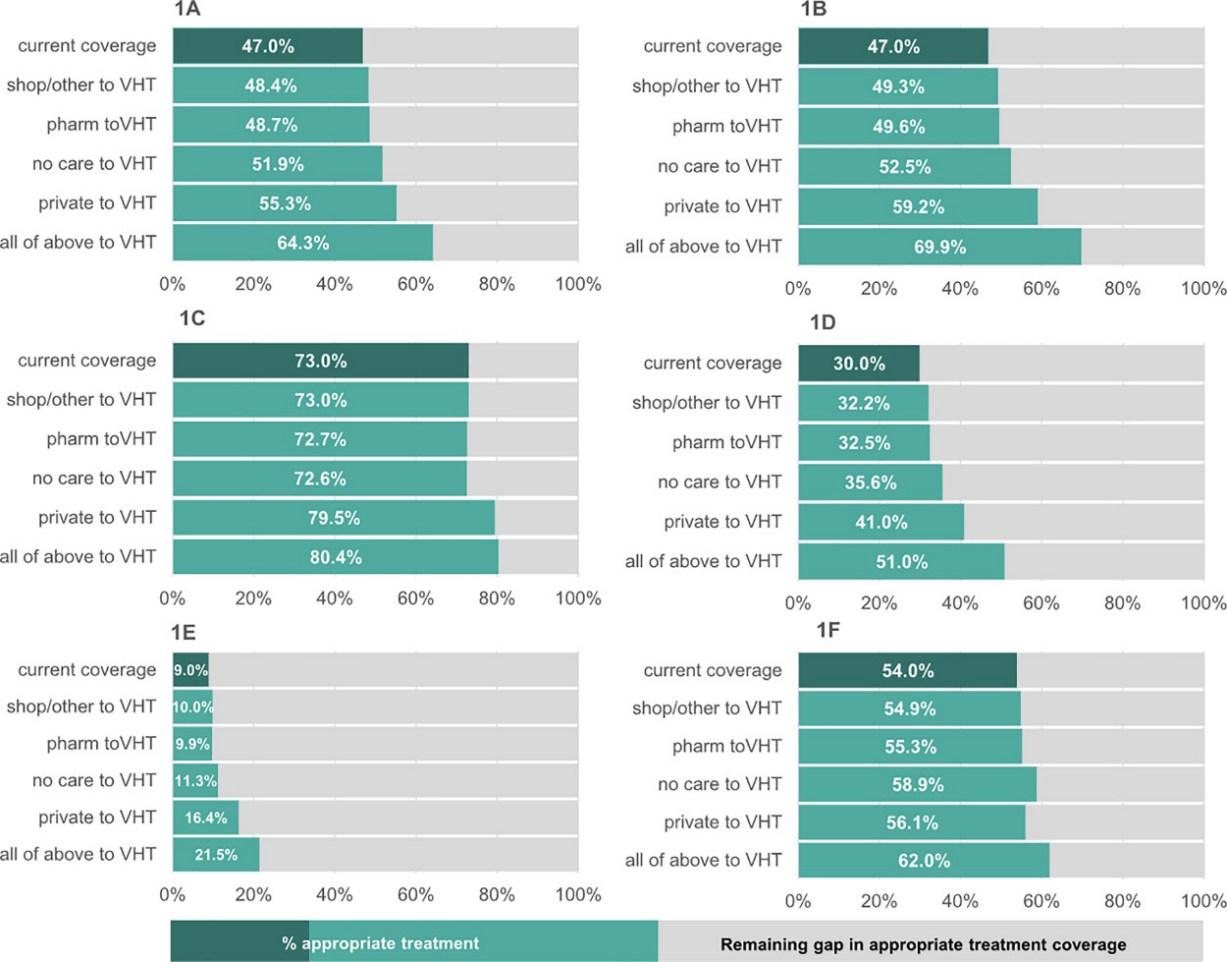
Predicted changes to the overall coverage of appropriate treatment for suspected/confirmed malaria, diarrhoea, and suspected pneumonia if caretakers of sick children switched from non-public sector providers or from not seeking care, to instead visiting the VHT as their first port of call for advice and/or treatment. Dark green bars show the current overall coverage rate of appropriate treatment for suspected/confirmed malaria, diarrhoea, suspected pneumonia or any episode of these. Lighter green bars show the predicted coverage rates of appropriate treatment if, all else being equal, caretakers currently using general shops, traditional healers or mobile services (OTHER), pharmacies (PHARM), private facilities or doctors (PRIVATE), not seeking care at all (NO CARE), or all those going to these providers combined (ALL OF ABOVE), switched to first using the VHT instead. 1a –all episodes of MDP; 1b –suspected malaria; 1c –confirmed malaria; 1d –diarrhoea (coverage of ORS treatment); 1e –diarrhoea (coverage of ORZ plus zinc treatment); 1f –suspected pneumonia.

Fig 1A illustrates that if all the children with MDP visiting non-public sector providers were first taken to the VHT instead, overall appropriate treatment would increase from 47% to 64%, or a reduction in the coverage gap from 53% to 36%. If children with suspected malaria currently not taken to public sector providers instead switched to visiting the local VHT, overall coverage of appropriate treatment could increase from 47% to 70% (reducing the gap from 53% to 30%, Fig 1B)–children with a confirmed diagnosis of malaria were more likely to receive the correct treatment irrespective of provider thus the improvement in overall coverage for confirmed malaria was smaller (+9.4%, Fig 1C). The same pattern of improvements was observed in children presenting with diarrhoea; resulting in an increase in diarrhoea appropriate treatment from 30% to 51% (Fig 1D; or an increase from 9% to 21.5% when restricted to those receiving both ORS and zinc tablets–Fig 1E), and for children with pneumonia where a more modest increase in appropriate treatment coverage was observed, from 54% to 62% (Fig 1F). In all scenarios, the single provider that had the largest impact on improving final treatment coverage after switching was the private doctor/facility, with the exception of those children with suspected pneumonia where those not seeking care at all had the largest impact on improving coverage of appropriate treatment.

The implications of this improved appropriate treatment coverage are outlined in Table 10, which shows the estimated overall percentage of sick children that would seek care to the VHT after switching from seeking care at the alternative provider specified in the list in the column farthest to the left. Switching from non-public provider care seeking to VHT care seeking increased overall VHT care seeking from the current rate of 20% of children, to a maximum rate of 77% of all children when all children outside the public sector switched (overall VHT care seeking figures are based on care seeking per child, and not per episode). This pattern was consistent across illness type.

**Table 10:** Updated percentages of children with suspected malaria, diarrhoea or suspected pneumonia who would seek care to the VHT (*as the first port of call), if children currently taken to the alternative providers listed on the left were instead taken to their VHT.

Current care seeking location* to switch from	Updated percentages of children seeking care to the VHT after switching, by condition				
	All episodes of MDP	Suspected malaria	Confirmed malaria	Diarrhoea	Suspected Pneumonia
% seeking care to VHT currently	20%	19%	40%	19%	21%
Shop/other to VHT	26%	25%	43%	25%	27%
Pharmacy to VHT	27%	27%	41%	25%	28%
No care to VHT	31%	29%	41%	30%	30%
Private sector to VHT	52%	52%	59%	54%	55%
All of the above to VHT	76%	76%	63%	77%	77%

## Discussion

Our study strongly indicates that iCCM trained health workers, if used, provide affordable, appropriate treatment for common childhood illnesses. Caretakers of sick children with suspected malaria, diarrhoea or pneumonia whose first port of call was the local iCCM-trained VHT in Uganda were more likely to receive appropriate treatment for their child in comparison to those who first visited any other health provider.

In addition to this, the out-of-pocket cost of seeking care to the VHT was the lowest of all providers, taking into account both direct medical costs and costs of transport in relation to care seeking. The body of evidence demonstrating that trained community health workers can successfully manage childhood illnesses in the community is growing [33–37]. However, to our knowledge, ours is the first study to report in detail the relative health and financial benefits to families of a scaled iCCM programme to fill the gap in the coverage of appropriate treatment for these most common childhood illnesses and to quantify these in comparison to existing health providers; results which are in clear agreement with the WHO/UNICEF statement in support of iCCM [6].

### Care seeking and cost of care

Despite the relative benefits to the care seeker in consulting the VHT, the VHT was the least used health provider in our survey, with only a fifth of children being taken to a VHT as the first port of call. The private health care sector, while often the most expensive option, was the least likely to result in proper case management–yet private providers were the most popular option in terms of health care seeking. All the children in our survey were from communities with at least one iCCM-trained VHT (the majority had two) who were identified and interviewed in a separate survey over the same time period as this study [20], hence seeking care to the VHT technically would have been a possible option for at least some children who were taken to the private sector, depending on the availability of the VHT during the illness episode in question. Families in our survey cited the perceived lack of drugs at the VHT and non-availability of the VHT as the main reasons for not using the service. Issues with staff workload, availability and stock supply are not uncommon given the setting, and point to the enduring barriers of CHW programmes [17]. These findings are in line with other studies of rural poor communities in Uganda and beyond [38–44]. In many such contexts, the number of private facilities outnumber public facilities and are more accessible to those in rural areas [18]. In addition, government investment in the public health service in Uganda and other similar sub-Saharan settings, though improving, has historically been inadequate, resulting in the continued perception of poorly equipped facilities and laboratories, and shortages of skilled staff–together resulting in the continued usage of private clinics and pharmacies even when public, cheaper consultations are available [38].

Families of sick children who were first taken to the VHT were most likely to then seek further advice or treatment at facility-based providers (particularly private providers) and pharmacies, which slightly increased the overall out-of-pocket cost of treating an illness episode whilst improving their overall likelihood of going on to receive correct treatment, if that treatment was not available at the VHT. Although we did not collect data on the reasons for further care seeking in those initially visiting their VHT, evidence in this context suggests that the most likely reasons are lack of availability of drugs at the VHT–and indeed at public facilities, thus driving families to the private sector [45]. Nonetheless the total costs to those who went elsewhere after visiting the VHT were still lower than the costs incurred by those who first visited private facilities and pharmacies. A recent study in the eastern part of Uganda similarly found that families accessing VHTs for children with fever or pneumonia incurred slightly lower out-of-pocket costs than those taken to facilities, although this study did not disaggregate public or private facilities, or include non-facility-based care seeking [46]. At the other end of the spectrum, the most expensive care seeking options for households in our survey were secondary (level 4/hospital) public facilities and private facilities (if used as the first port of call)–resulting in out-of-pocket costs sometimes in excess of USD 25. Although health care for children under five years of age is free of charge at level 4+ public facilities, we have shown that non-medical costs including travel and subsistence can contribute considerably to the overall outlay. The 2009–2010 Ugandan census estimated the mean monthly income from all sources (cash and in-kind) for a rural household in Uganda at USD 90 [47] though over half of households in our survey reported a monthly cash income of less than USD 20, meaning care-seeking costs higher than USD 25 may constitute a significant cost burden for some families. Despite the availability of free care through the iCCM VHT service in all the communities surveyed, 21% of families who did not seek care outside the home still cited cost as the main reason for this, indicating that even the risk of incurring non-direct costs can be prohibitive in the care seeking process as has been indicated in other studies [45].

### Coverage gaps in appropriate treatment

Notably, this study demonstrates an enduring and significant gap in coverage of appropriate treatment for sick children under 5 years of age, within the context of a working iCCM programme. More than 50% of cases of MDP were not treated appropriately, with the largest gaps observed for ORS and zinc treatment in children with diarrhoea. Whilst we have shown that those taken to the VHT received better than average care across the board, gaps remain even in this group. It is important to note that the iCCM-VHT programme was relatively new in this area at the time of the survey and some improvement will have occurred since then as functionality increases. Our findings nonetheless echo recent research in the region showing that whilst iCCM programme implementation can improve access to appropriate treatment for sick children, coverage of treatment is often far from adequate [48–50]. The reasons for these gaps are myriad and often complex. In our setting, we found that failure to seek care at all, which itself has many causes ranging from socio-economic, geographical to behavioural [28, 29], was a key driver for failure to receive any drugs (S3 Table) additionally, some children instead received drugs that were not/no longer recommended for their condition (S3 Table). Lack of stock has been an observed barrier to appropriate treatment for malaria and pneumonia in similar settings [51], and will likely have been a contributing factor here [45]. Incorrect symptom management may also occur as a result of misdiagnosis of suspected malaria and pneumonia especially when no tests are available [52, 53].

The case of ORS and zinc warrants special exploration. The scientific evidence for the ability of ORS and zinc supplementation to reduce the duration and deaths associated with acute childhood diarrhoea is robust [54, 55], and is a management strategy that is strongly endorsed by the WHO and UNICEF [56]. A position paper in the Bulletin of the WHO now written nearly 10 years ago noted that coverage of ORS and zinc in the treatment of diarrhoea was low despite this evidence and despite expert recommendation—and urged a renewed call to action. Global coverage then stood at 38%, which is 8% higher than we observed in this study [57]. Reasons cited in the position paper and elsewhere include the limited global supply of the correct formulations, lack of financing, poor national promotion/demand resulting in lack of availability at the primary care level (especially the private sector), and the low priority of ORS and zinc treatment versus other interventions on the international public health stage as main causes [57–59]. The results of our survey and evidence from more recent surveys also reporting inadequate treatment coverage or prescribing practices for ORS/zinc in Uganda [60, 61] suggests that little had changed.

### Use of population-based household survey data

Our findings are centred on caregiver reports of the child’s symptoms and of treatment received, using questions based on validated United Nations- and USAID-supported Multiple Indicator Cluster Survey and Demographic Health Surveys tools. These systems use population-survey techniques to provide indicators for a range of public health and socio-economic outcomes to inform and or monitor programmes and policy on both national and international scales [21, 22, 43]. The socio-demographic profile of our study participants indicates a rural, farming population the majority who are under 30 years of age, and for those who have any formal education, who leave the educational system in childhood or adolescence. Given that i) our sampling strategy was based on the random selection of communities and households within the study site and ii) experiences of the authors and others suggests that participation rates are high amongst those invited for interview (in a follow-up household survey we conducted of child health and treatment in the same study site in 2015 the non-availability/refusal rate of caretakers was 0.5%) we have no reason to believe that our sample deviates in any significant way from the profile of the wider study population. Evidence from studies in the region and from Uganda census data for Bunyoro (the state in which our study site lies) confirms a similar demographic profile with average household incomes well under $20/month [34, 47, 50, 62]. Nonetheless, we acknowledge that caregiver recall of symptoms and treatments received will be subject to a level of measurement error, which will impact the accuracy of estimates of disease prevalence and appropriate treatment rates, particularly for malaria and pneumonia, whose symptom patterns show some overlap [63]. Furthermore the possibility of co-morbidities in this setting is high [63] therefore we did not assume a hierarchy for condition diagnosis, though clinicians/health workers in our study area may have done so. It is possible that the prevalence of illness and particularly of malaria and pneumonia may be overestimated by using population-survey techniques despite validation, thus underestimating the overall coverage of appropriate treatment. A group of seminal studies assessed the quality of caregiver-reported signs, symptoms and treatment of MDP in comparison to clinical diagnosis or observations of clinic visits (the “gold standards”). They recorded sensitivities ranging from 31%-81% and specificities of 38%-91% for the prevalence and treatment of these conditions in a variety of settings [64–66]. The authors demonstrated that i) diagnoses based on fewer symptoms (e.g. cough and fast breathing/chest indrawing only for suspected pneumonia) inflate estimates of the true rates with false positives, ii) accuracy of estimates could be increased by using pill boards to assist in treatment recall, video examples of children with the disease in question, including more symptoms (though not indiscriminately) in definitions of disease, and/and longer recall periods [64–66]. By the use of drug cards (S1 Fig), a large sample size, and local dialect equivalents of symptom names, we endeavoured to improve the accuracy of caregiver recall and thus of estimates of symptoms and treatments in our study; even so it is not possible to eliminate this error entirely. In particular our definition of appropriate treatment for malaria included cases of presumptively treated fever where no test was available; this was necessary in order to reflect the clinical guidelines in Uganda at the time [67]. Though policy has since changed [68], our estimates of suspected malaria will include false positives–which were still treated ‘appropriately’ in accordance with the 2010 guidelines. Also, while fever, diarrhoea and pneumonia are amongst the most common causes of illness in children under five in the region [5, 69–73], it is possible that some of the children in the survey with MDP also had another co-morbidity; this may have shifted care-seeking costs upwards if treatment was also given for co-morbidities not captured within the range of symptoms asked for. Another limitation with caregiver recall is the placing of the illness episode within the recall period, although it might have occurred before, and not remembering exact expenditures amounts which occurred some days back in time.

Nonetheless ultimately and most importantly, the aim of this paper is to contrast the quality of the available providers in the treatment of children, thus its internal validity still holds [65]. We observed consistent, large and often significant differences between providers, including in the subsample of children with a blood test-confirmed diagnosis of malaria (the recall of such tests of which has been shown to be highly specific [64]), with the same outcome: the VHT, followed by the public sector provided the best care.

#### Intra-class correlation coefficients for appropriate treatment coverage of children under 5 years of age

The clustering of population survey data is a common feature particularly of large studies implemented at scale. We calculated the cluster ICCs for appropriate treatment, which indicated that appropriate treatment outcomes for malaria was more likely to cluster within communities than treatment for other conditions, with pneumonia treatment the least likely. As ICCs (or the related design effect) are required to estimate sample sizes for the evaluation of interventions and programmes, we hope that future trials reporting on population-level treatment outcomes will find these useful. To our knowledge, this is the first time that ICCs for community-level clustering of appropriate treatment for MDP have been presented; we have found only studies systematically reporting treatment-related ICCs for facility or health-worker clusters [74–76]. ICCs for the appropriate treatment of MDP are particularly useful in the present context where there is increasing interest in understanding the effectiveness of iCCM programmes and their scale-up [77]. Note the applicability of our ICCs could extend beyond iCCM to any programmes which investigate similar child health outcomes and/or treatments, and for which ICC data is otherwise difficult to obtain.

### Increasing rates of care seeking to the iCCM CHW–impact on coverage of appropriate treatment

Less than half of 5057 episodes of MDP in children in our survey were appropriately treated. We estimated that coverage of appropriate treatment could increase to nearly two thirds overall (64%)–a gain of 17 percentage points–if sick children visited the VHT instead of private providers or instead of not seeking care, though larger improvements in coverage were estimated for some individual conditions. Although we did not collect data on the length of time between onset of symptoms and the start of treatment with suitable drugs, the VHT, being based within rural communities with otherwise poor access to health clinics, may on average also increase the timeliness of appropriate treatment of children who might otherwise need to travel some distance to the nearest alternative provider [6], and indeed the communities targeted for the government VHT programme were selected based on this criteria [78]. Thus, an added benefit of increased VHT care seeking at the expense of private sector care or no care seeking, even if not to the degree demonstrated in our exercise, is the potential increase in coverage of not only appropriate but also prompt treatment.

Despite these advantages, the estimated VHT care seeking rates that would occur if children switched from care seeking in the non-public sector rose to a peak of 77% of all sick children–a more than three-fold increase in the current rate of 20% seeking care at the VHT. Putting aside the feasibility of a transformation in family care seeking behaviours on this scale, such an increase may nonetheless constitute a massive and likely unsustainable burden on some iCCM programmes. Scale-up of iCCM in sub-Saharan Africa already suffers from a range of chronic bottlenecks including sub-optimal health systems infrastructure, unreliable drug supply, inadequate community buy-in, and poor motivation and retention of CHWs amongst other barriers [17, 78, 79]. In a recent review [80] of iCCM programmes in 10 countries in sub-Saharan Africa it was estimated that CHWs conducted between 1–76 treatments per month (median = 11). Nevertheless, is not possible to state with certainty whether the programme in mid-Western Uganda could sustain up to a three-fold increase in care seeking rates; at the time of our survey (2011), the Uganda Ministry of Health had shared a Strategic Plan to increase the number of iCCM-trained VHTs per village to 5 per village with 4 out of 5 trained by 2015 [78]. This in itself is still not enough to encourage and sustain an increase in care seeking; however, if key associated systems (CHW supervision and training, equipment and commodity supply chain) were also successfully scaled up, and strategies to increase demand-side support and utilisation of iCCM were implemented, then we see no reason why the appropriate treatment coverage gap could not be reduced on a scale similar to that which is projected in this paper.

The reality is however, that it will be likely that some will continue to use private sector providers as the first port of call, and indeed there may be good economic reasons to maintain the sector [81]. The training of drug shops and private doctors/clinics to diagnose and treat MDP according to recommended guidelines will therefore be important additive or alternative channel to reducing the treatment gap, particularly given the popularity of the private sector and its significant influence on overall coverage. Two recent studies reported varying impact on appropriate treatment of MDP following training and supply of private sector providers [82, 83], though there are yet few comprehensive, national strategies or programmes that cover this sector [84]. Ultimately, both increasing utilisation of iCCM services in coordination with support and training to drug shops and private providers may be viewed as complimentary processes by which health ministries in low income settings can continue to reduce treatment coverage gaps [81].

## Conclusions

Universal health coverage is a vital stepping stone to achieving equity in treatment quality for the global community [8]. With this paper, we have added key health provider performance and demand-side costs data to a growing body of programme evaluation evidence demonstrating that iCCM-trained community health worker programmes have the potential to perform a crucial role in this system through reducing the gap in the appropriate treatment of sick children. It also highlights the particular benefit of such programmes to the rural poor in low and middle-income settings. There is now scope for additional studies to unpack the impact of this programme amongst important subgroups including by the gender and/or age of child, and by the relative wealth of households. Ultimately, continued national and international support for the scale up of iCCM programmes, combined with increased user buy-in are imperative steps to achieving and sustaining health equity goals.

## Supporting information

S1 Table Definitions of illness types, care providers and appropriate treatment. **1a - Definitions of common childhood illnesses.** Definitions of diagnosis and treatment are based on standard UN guidelines for treatment of malaria, diarrhoea and pneumonia in the community and at health facilities [10, 23–26]; **1b - Definitions of health provider types; 1c** - **Definitions of appropriate treatment.** Definitions of diagnosis and treatment are based on standard UN guidelines for treatment of malaria, diarrhoea and pneumonia in the community and at health facilities [10, 23–26]; **1d** - **Definitions of medical and non-medical care seeking costs.**
(DOCX)

S2 Table Care seeking patterns of children with a fever (including fevers which turned out to be malaria negative), those with fever who received a malaria blood test, and children with a confirmed malaria diagnosis. (DOCX)

S3 Table Outcomes of 2695/5057 episodes of suspected malaria, diarrhoea or suspected pneumonia that did not receive the appropriate treatments. * Received any drug not included in national/international guidelines for treatment of the specific condition (episodes of suspected malaria, diarrhoea or suspected pneumonia)—includes unknown drugs not listed in survey, where caretaker nonetheless reported that treatment was given.
(DOCX)

S4 Table Intra-class correlation coefficients (ICC) of the percentage of children with suspected/confirmed malaria, diarrhoea, suspected pneumonia, and of all episodes of MDP who were appropriately treated within 41 sub-counties (the cluster identifier) in mid-Western Uganda 2011. ICCs calculated using the unweighted analysis of variance estimator (‘loneway’ command) in Stata 13.1: This calculates an ICC as a function of the F-statistic from a one way analysis of variance of appropriate treatment rate with cluster ID as the only predictor.
(DOCX)

S5 Table Itemised and total mean costs of seeking care for children with an episode of MDP, USD 2011 stratified by the first location visited ^a^7 records dropped due to abnormally high costs (4 records) or missing cost data (3 records); *SD standard deviation; Note public facilities are disaggregated by primary (level II or III) or secondary (level IV or hospital) care levels as costs were accrued differently between the two. (DOCX)

S6 Table Medical, non-medical and total costs of seeking care at the first location visited by children with an episode of MDP, USD 2011 stratified by the first location visited. **Public facilities are disaggregated by primary (level II or III) or secondary (level IV or hospital) care levels as costs were accrued differently between the two.** ^a^7 records dropped due to abnormally high costs (4 records) or missing cost data (3 records); *IQR interquartile range, SD standard deviation; ** registration fees, medicines, consumables, 'gratuities'; ***transport, subsistence **costs.**
(DOCX)

S1 Questionnaire inSCALE cross-sectional baseline survey household and sick child instrument. (PDF)

S1 Fig inSCALE cross-sectional baseline survey drug picture cards. (PDF)
