# Supplementary material for: Variation in the quality and out-of-pocket cost of treatment for childhood malaria, diarrhoea, and pneumonia: Community and facility based care in rural Uganda
Source: PLoS One. 2018 Nov 26;13(11):e0200543. doi: 10.1371/journal.pone.0200543 (PMC6261061; doi:10.1371/journal.pone.0200543)
Supplement: S1 Table — 1a - Definitions of common childhood illnesses. Definitions of diagnosis and treatment are based on standard UN guidelines for treatment of malaria, diarrhoea and pneumonia in the community and at health facilities [10, 23–26]; 1b - Definitions of health provider types; 1c - Definitions of appropriate treatment. Definitions of diagnosis and treatment are based on standard UN guidelines for treatment of malaria, diarrhoea and pneumonia in the community and at health facilities [10, 23–26]; 1d - Definitions of medical and non-medical care seeking costs. (DOCX) [file pone.0200543.s001.docx]

# Supporting Information table 1

Definitions of the prevalence of illness, care seeking, appropriate treatment and out of pocket costs accompanying manuscript “Variation in the quality and out-of-pocket cost of treatment for childhood diarrhoea, malaria and pneumonia: community and facility based care in rural Uganda. Soremekun et al 2017”

## S1a Table. Definitions of common childhood illnesses

| **Disease category** | **Symptoms reported by caregivers** | **Age range of child** |
| --- | --- | --- |
| Suspected malaria | Fever symptoms in previous two weeks. Excluding those children with fever confirmed negative for malaria via blood test | 4 months to 59 months of age |
| Confirmed malaria | Fever plus a blood test positive for malaria | 4 months to 59 months of age |
| Diarrhoea | Diarrhoea in the previous two weeks. Passed 3 or more watery stools in a 24 hour period not containing blood or mucus. | 2 months to 59 months of age |
| Suspected pneumonia | Cough with fast or difficult breathing in previous two weeks, or chest indrawing. | 2 months to 59 months of age |
| Any of suspected malaria, diarrhoea or suspected pneumonia (MDP) | Any child with symptoms conforming to any of the above illnesses | 2 months to 59 months of age |

## S1b Table. Definitions of health provider types

| **Care seeking category** | **Included types of provider** |
| --- | --- |
| Village Health Team member (VHT) | iCCM-trained VHT |
| Public facility | A state-subsidised clinic or hospital (clinic levels HC2-HC4 and hospital) |
| Private facility | A private clinic, hospital or doctor not subsidised by the state |
| Pharmacy | A pharmacy or drug shop |
| General shop/other | General (convenience) shop, traditional healer, mobile services |
| No care sought | No care sought outside the home/family |

## S1c Table. Definitions of appropriate treatment

| **Disease category** | **Appropriate treatments** |
| --- | --- |
| Suspected malaria | Artemether-lumefantrine, dihydroartemisinin-piperaquine, amodiaquin-artesunate, or artesunate-sulfadoxine-pyrimethamine. If any danger sign is reported alongside a diagnosis of suspected malaria: rectal artesunate |
| Confirmed malaria | As above |
| Diarrhoea | ORS, or homemade rehydration solution taken. ORS plus oral zinc supplementation analysed as a secondary indicator. |
| Suspected pneumonia | Amoxycillin, chloramphenicol, erythromycin, feftriaxone, azythromycin, gentamycin, or cephalexin taken |
| Any of suspected malaria, diarrhoea or suspected pneumonia (MDP) | Received the correct treatments (as above) specific to any case of MDP |

Definitions of diagnosis and treatment are based on standard UN guidelines for treatment of malaria, diarrhoea and pneumonia in the community and at health facilities [1-5].

## S1d Table. Definitions of out of pocket costs

| **Cost category** | **Type of costs** |
| --- | --- |
| Direct medical costs | Patient registration fees, medicines, material costs (like gloves, iv, syringes etc.), monetary gifts/contributions/ gratuities to health providers |
| Direct non-medical costs | Transportation, sustenance costs (e.g. food, drinks), accommodation. |

**Refs**

1. UNICEF. Health: Definitions of indicators <https://www.unicef.org/infobycountry/stats_popup3.html> (accessed 10 August 2017): UNICEF; 2004.

2. World Health Organisation. Guidelines for the treatment of malaria. Geneva, Switzerland: World Health Organisation, 2015.

3. World Health Organisation. Manual for the Community Health Worker: Caring for the sick child in the community - treat diarrhoea, confirmed malaria and fast breathing. Geneva, Switzerland: WHO, 2011.

4. World Health Organisation. Revised WHO classification and treatment of pneumonia in children at health facilities: evidence summaries. Geneva, Switzerland: WHO, 2014.

5. World Health Organisation. Handbook IMNCI: Integrated Management of Childhood Illness. Geneva, Switzerland: WHO, 2005.
