# Supplementary material for: Variation in the quality and out-of-pocket cost of treatment for childhood malaria, diarrhoea, and pneumonia: Community and facility based care in rural Uganda
Source: PLoS One. 2018 Nov 26;13(11):e0200543. doi: 10.1371/journal.pone.0200543 (PMC6261061; doi:10.1371/journal.pone.0200543)
Supplement: S2 Table — (DOCX) [file pone.0200543.s002.docx]

# Supporting Information table 2

**S2 Table**. Care seeking patterns of children with a fever (including fevers which turned out to be malaria negative), those with fever who received a malaria blood test, and children with a confirmed malaria diagnosis.

| Location | Fever (n=2921) |
| --- | --- |
|  | % (n) |
| VHT | 20% (591) |
| Public facility | 25% (735) |
| Private facility/doctor | 32% (926) |
| Pharmacy | 7% (212) |
| General shop/other | 6% (174) |
| No care sought | 10% (283) |
