# Supplementary material for: Variation in the quality and out-of-pocket cost of treatment for childhood malaria, diarrhoea, and pneumonia: Community and facility based care in rural Uganda
Source: PLoS One. 2018 Nov 26;13(11):e0200543. doi: 10.1371/journal.pone.0200543 (PMC6261061; doi:10.1371/journal.pone.0200543)
Supplement: S3 Table — * Received any drug not included in national/international guidelines for treatment of the specific condition (episodes of suspected malaria, diarrhoea or suspected pneumonia)—includes unknown drugs not listed in survey, where caretaker nonetheless reported that treatment was given. (DOCX) [file pone.0200543.s003.docx]

# Supporting Information table 3

**S3 Table.** Outcomes of 2695/5057 episodes of suspected malaria, diarrhoea or suspected pneumonia that did not receive the appropriate treatments

| **1^st^ provider at which care sought** | **% No drugs received at all (n)** | **% Received other drugs (n)** |
| --- | --- | --- |
| **Overall** | **17% (452)** | **83% (2243)** |
| VHT | 8% (28) | 92% (317) |
| Public health facility | 8% (39) | 92% (426) |
| Private health facility/doctor | 6% (62) | 94% (964) |
| Pharmacy | 3% (6) | 97% (224) |
| General shop/other | 6% (11) | 94% (183) |
| No care sought | 70% (306) | 30% (129) |

* Received any drug not included in national/international guidelines for treatment of the specific condition (episodes of suspected malaria, diarrhoea or suspected pneumonia) - includes unknown drugs not listed in survey, where caretaker nonetheless reported that treatment was given.
