# Supplementary material for: Variation in the quality and out-of-pocket cost of treatment for childhood malaria, diarrhoea, and pneumonia: Community and facility based care in rural Uganda
Source: PLoS One. 2018 Nov 26;13(11):e0200543. doi: 10.1371/journal.pone.0200543 (PMC6261061; doi:10.1371/journal.pone.0200543)
Supplement: S4 Table — ICCs calculated using the unweighted analysis of variance estimator (‘loneway’ command) in Stata 13.1: This calculates an ICC as a function of the F-statistic from a one way analysis of variance of appropriate treatment rate with cluster ID as the only predictor. (DOCX) [file pone.0200543.s004.docx]

# Supporting Information table 4

**S4 Table.** Intra-class correlation coefficients (ICC) of the percentages of children with suspected/confirmed malaria, diarrhoea, suspected pneumonia, and of all episodes of MDP who were appropriately treated within 41 sub-counties (the cluster identifier) in mid-Western Uganda 2011.

| **Suspected malaria** | **Confirmed malaria** | **Diarrhoea (ORS)** | **Diarrhoea (ORS+zinc)** | **Pneumonia** | **All episodes** |
| --- | --- | --- | --- | --- | --- |
| 0.0328 | 0.0459 | 0.0371 | 0.0203 | 0.0167 | 0.0206 |

ICCs calculated using the unweighted analysis of variance estimator (‘loneway’ command) in Stata 13.1: This calculates an ICC as a function of the F-statistic from a one way analysis of variance of appropriate treatment rate with cluster ID as the only predictor [1, 2].

**Refs**

1. Kish L. Survey Sampling: Wiley-Blackwell; 1995. 664 p.

2. StataCorp LP. Loneway - Large one-way ANOVA, random effects, and reliability. Stata Reference Manual Release 13. College Station, Texas: Stata Press; 2013. p. 1096-101.
