# Supplementary material for: Variation in the quality and out-of-pocket cost of treatment for childhood malaria, diarrhoea, and pneumonia: Community and facility based care in rural Uganda
Source: PLoS One. 2018 Nov 26;13(11):e0200543. doi: 10.1371/journal.pone.0200543 (PMC6261061; doi:10.1371/journal.pone.0200543)
Supplement: S5 Table — (DOCX) [file pone.0200543.s005.docx]

# Supporting Information table 5

**S5 Table.** Itemised and total mean costs of seeking care for children with an episode of MDP, USD 2011 stratified by the first location visited

| First care seeking location | N^a^ | Out-of-pocket costs in relation to seeking care for most recent illness episode  (including costs of up to two care seeking locations) | | | | | | |
| --- | --- | --- | --- | --- | --- | --- | --- | --- |
|  |  | Mean (SD*) | | | | | | |
|  |  | Medical | | | | Non-medical | | Total expenses |
|  |  | Registration fees/ clinical charges | Medicines | Consumables | 'Gratuities' | Transport | Subsistence |  |
| VHT (public sector level I) | n=662 | 0.03 (0.36) | 1.24 (4.27) | 0.02 (0.17) | 0.02 (0.21) | 0.21 (0.98) | 0.50 (1.71) | 2.0 (5.6) |
| Public health facility, primary care only (public sector level II or III) | n=655 | 0.02 (0.14) | 1.60 (4.97) | 0.14 (1.47) | 0.21 (3.40) | 0.36 (1.17) | 0.57 (2.25) | 2.9 (10.2) |
| Public health facility, with inpatient care (public sector level IV or hospital) | n=143 | 0.06 (0.31) | 2.58 (6.20) | 0.15 (0.66) | 0.29 (3.36) | 1.25 (3.35) | 1.36 (4.68) | 5.7 (13.7) |
| Private health facility (clinic or hospital) or doctor | n=1,059 | 0.06 (0.90) | 3.59 (4.64) | 0.14 (0.96) | 0.09 (1.08) | 0.45 (2.43) | 0.86 (3.11) | 5.2 (8.4) |
| Private pharmacy | n=255 | 0.01 (0.12) | 1.77 (2.44) | 0.01 (0.07) | 0.00 (0.00) | 0.12 (0.58) | 0.21 (0.98) | 2.1 (2.9) |
| General shop/other | n=202 | 0.04 (0.24) | 2.71 (4.12) | 0.06 (0.26) | 0.05 (0.36) | 0.22 (0.78) | 0.52 (1.36) | 3.6 (5.2) |

^a^7 records dropped due to abnormally high costs (4 records) or missing cost data (3 records); *SD standard deviation; Note public facilities are disaggregated by primary (level II or III) or secondary (level IV or hospital) care levels as costs were accrued differently between the two.
