# Supplementary material for: Variation in the quality and out-of-pocket cost of treatment for childhood malaria, diarrhoea, and pneumonia: Community and facility based care in rural Uganda
Source: PLoS One. 2018 Nov 26;13(11):e0200543. doi: 10.1371/journal.pone.0200543 (PMC6261061; doi:10.1371/journal.pone.0200543)
Supplement: S6 Table — Public facilities are disaggregated by primary (level II or III) or secondary (level IV or hospital) care levels as costs were accrued differently between the two. a7 records dropped due to abnormally high costs (4 records) or missing cost data (3 records); *IQR interquartile range, SD standard deviation; ** registration fees, medicines, consumables, 'gratuities'; ***transport, subsistence costs. (DOCX) [file pone.0200543.s006.docx]

# **Supporting Information table 6**

**S6 Table.** Medical, non-medical and total costs of seeking care at the first location visited by children with an episode of MDP, USD 2011 stratified by the first location visited. Public facilities are disaggregated by primary (level II or III) or secondary (level IV or hospital) care levels as costs were accrued differently between the two.

| First care seeking location | N^a^ | Out-of-pocket costs first point of care for most recent illness episode | | | | | |
| --- | --- | --- | --- | --- | --- | --- | --- |
|  |  | Median (IQR*) | | | Mean (SD*) | | |
|  |  | Medical** | Non-medical*** | Total expenses | Medical | Non-medical | Total expenses |
| VHT (public sector level I) | n=662 | 0 .0 (0.0-0.0) | 0.0 (0.0-0.0) | 0.0 (0.0-0.0) | 0.0 (0.5) | 0.3 (1.0) | 0.3 (1.2) |
| Public health facility, primary care only (public sector level II or III) | n=655 | 0.0 (0.0-0.0) | 0.0 (0.0-0.4) | 0.0 (0.0-1.2) | 1.0 (4.1) | 0.7 (2.0) | 1.7 (5.6) |
| Public health facility, with inpatient care (public sector level IV or hospital) | n=143 | 0.0 (0.0-2.0) | 0.4 (0.0-2.0) | 1.0 (0.0-4.3) | 2.6 (9.1) | 2.4 (6.5) | 5.1 (13.5) |
| Private health facility (clinic or hospital) or doctor | n=1,059 | 2.3 (0.8-4.4) | 0.0 (0.0-1.0) | 2.8 (1.0-6.0) | 3.7 (5.1) | 1.2 (4.0) | 4.9 (7.7) |
| Private pharmacy | n=255 | 1.0 (0.2-2.1) | 0.0 (0.0-0.0) | 1.0 (0.4-2.8) | 1.7 (2.4) | 0.3 (0.8) | 2.0 (2.7) |
| General shop/other | n=202 | 1.4 (0.2-3.2) | 0.0 (0.0-0.7) | 1.8 (0.2-4.1) | 2.6 (3.6) | 0.7 (1.6) | 3.3 (4.5) |

^a^7 records dropped due to abnormally high costs (4 records) or missing cost data (3 records); *IQR interquartile range, SD standard deviation; ** registration fees, medicines, consumables, 'gratuities'; ***transport, subsistence costs
