# Supplementary material for: Variation in the quality and out-of-pocket cost of treatment for childhood malaria, diarrhoea, and pneumonia: Community and facility based care in rural Uganda
Source: PLoS One. 2018 Nov 26;13(11):e0200543. doi: 10.1371/journal.pone.0200543 (PMC6261061; doi:10.1371/journal.pone.0200543)
Supplement: S1 Questionnaire — (PDF) [file pone.0200543.s007.pdf]

Supporting Information Questionnaire 1

S1 Questionnaire. inSCALE Cross-sectional baseline survey Household and Sick Child instrument.

Integrated Community Case Management of Childhood Diseases in Uganda

FORM ID LABEL HERE

INSCALE BASELINE HOUSEHOLD SURVEY 2011

Version 15/04/2011

COMPOUND AND INTERVIEW INFORMATION

	QUESTION	FORMAT	
1.1.	District name	text	____________________
1.2.	Subcounty Name	text	____________________
1.3.	LC1/Village name	text	____________________
1.4.	Interviewer's code	no.	☐☐
1.5.	Date of Interview	<DD/MM/20YY>	☐☐ / ☐☐ / 2 0 ☐☐
1.6.	Time started	Hrs: Min	☐☐ : ☐☐
		circle	AM 1<br>PM 2
1.7.	Time ended:	Hrs: Min	☐☐ : ☐☐
		text	AM 1<br>PM 2
1.8.	Written informed consent provided?	circle	Yes 1<br>No 2
1.9.	Household ID (to be filled by supervisor)		☐☐ . ☐☐ . ☐ . ☐☐
1.10	Was this interview completed?	circle	Yes 1<br>No 2

If no, explain why not in the comments box below

1.11 **Do you - the RA - have any comments regarding this visit/interview?**:

S2 SECTION 2 DEMOGRAPHICS AND SES

2.1.1	What is your name **[Official Name]**			______________________
2.1.2	Do you have another name by which people know you in this community? [Common Name]	If none given write NA		______________________
2.1.3	**[Observe sex of the respondent]**		Male	1
			Female	2
2.1.4	In what year were you born?	<YYYY>	Don’t Know=9999	☐☐☐☐
2.1.5	In what month were you born?		January	1
			February	2
			March	3
			April	4
			May	5
			June	6
			July	7
			August	8
			September	9
			October	10
			November	11
			December	12
			Don’t Know	99
2.1.6	What is your age?	number	Don’t Know=999	☐☐☐
2.1.7	What is your relation to the head of the household		I am head of household	1
			Wife or husband	2
			Son or daughter	3
			Son-in-law or daughter-in-law	4
			Grandchild	5
			Parent	6
			Parent-in-law	7
			Brotheror Sister	8
			Niece/nephew by blood	9
			Niece/nephew by marriage	10
			Otherrelative	11
			Adopted/foster/stepchild	12
			Not related	13
			Don't know	99

form ID = ☐☐☐☐☐☐☐

2.1.8	What is your mother tongue	Acholi	1	→ 2.1.10
		Alur	2	
		Lugisu	3	
		Luganda	4	
		Lugbara	5	
		Rugungu	6	
		Rukiga	7	
		Runyarwanda	8	
		Runyoro	9	
		Rutoro	10	
		Runyankole	11	
		Other	88	
2.1.9	**[If other:]** Specify	______________________________		
2.1.10	What is your religion?	None, don't have one	1	→ 2.1.12
		Christian (Protestant)	2	
		Christian (Roman Catholic)	3	
		Christian (Pentecostal)	4	
		Christian (Seventh Day Adventist)	5	
		Muslim	6	
		Traditional	7	
		Faith of Unity	8	
		Other	88	
2.1.11	**[If other:]** Specify	______________________________		
2.1.12	What is your principal occupation?	Employed in private sector or by NGO	1	→ 2.1.14
		Civil servant	2	
		Casual worker (manual/agriculture)	3	
		Self-employed farmer/ peasant/fisherman/cattlekeeper	4	
		Self-employed business man/women	5	
		Domestic work in own household	6	
		Unemployed (looking for work)	7	
		Unemployed (not looking for work)	8	
		Sick/disabled and unable to work	9	
		Student	10	
		Other	88	
2.1.13	**[If other:]** Specify	______________________________		
2.1.14	Did you attend school? [Also answer Yes if respondent is still in school]	Yes	1	
		No	2	→ 2.1.16

2.1.15 [If Yes:] until which grade/class did you attend? circle

Primary	1	2	3	4	5	6	7	8
Secondary	1	2	3	4	5	6		
Tertiary	1	2	3	4	5			
Vocational	1	2	3	4				

2.1.16 Can you read and understand a letter or newspaper, easily, or with difficulty, or not at all, in any language?

Yes, I can easily read without any difficulty	1
Yes, I can read but with some difficulty	2
No, I cannot read at all	3

2.1.17 Can you write a letter easily, or with difficulty, or not at all, in any language?

Yes, I can write easily without difficulty	1
Yes, I can write with some difficulty	2
No, I cannot write at all	3

[<u>CHECK</u> with Q2.1.7]
[If the respondent is the head of household:] SKIP to question: 2.3.1
[If the respondent is not the head of household, say:]

Now I will ask some questions about the head of this household

2.2.1 What is the name of the head of household? **[Official Name]** ______________________________

2.2.2 Does the head of household have another name by which people know him in this community? [Common Name] ______________________________

2.2.3 Is the head of household Male or Female?

Male	1
Female	2

2.2.4 In what year was the head of household born? <YYYY> Don't Know=9999 [ ][ ][ ][ ]

2.2.5 In what month was the head of household born?

JANUARY	1
FEBRUARY	2
MARCH	3
APRIL	4
MAY	5
JUNE	6
JULY	7
AUGUST	8
SEPTEMBER	9
OCTOBER	10
NOVEMBER	11
DECEMBER	12
DON'T KNOW	99

2.2.6 What is the age of the head of household? Don't Know=99 [ ][ ][ ]

form ID = ☐☐☐☐☐☐☐

2.2.7	What is the mother tongue of the head of this household?	Acholi	1 → 2.2.9
		Alur	2
		Lugisu	3
		Luganda	4
		Lugbara	5
		Rugungu	6
		Rukiga	7
		Runyarwanda	8
		Runyoro	9
		Rutoro	10
		Runyankole	11
		Other	88
2.2.8	**[If other:]** Specify	______________________________	
2.2.9	What is the religion of the head of household?	None, doesn't have one	1 → 2.2.11
		Christian (Protestant)	2
		Christian (Roman Catholic)	3
		Christian (Pentecostal)	4
		Christian (Seventh Day Adventist)	5
		Muslim	6
		Traditional	7
		Faith of Unity	8
		Other	88
2.2.10	**[If other:]** Specify	______________________________	
2.2.11	What is the principal occupation of the head of household?	Employed in private sector or by NGO	1 → 2.2.13
		Civil servant	2
		Casual worker (manual/agriculture)	3
		Self-employed farmer/ peasant/fisherman/cattlekeeper	2
		Self-employed business man/women	3
		Domestic work in own household	4
		Unemployed (looking for work)	5
		Unemployed (not looking for work)	6
		Sick/disabled and unable to work	7
		Student	8
		Other	88
2.2.12	**[If other:]** Specify	______________________________	
2.2.13	Can the head of household read and understand a letter or newspaper, easily, or with difficulty, or not at all, in any language?	Yes, head of household can easily read without any difficulty	1
		Yes, the head of household can read but with some difficulty	2
		No, the head of household cannot read at all	3
		Don't know	99

2.2.14 Can the head of household write a letter easily, or with difficulty, or not at all, in any language?

Yes the head of household can write easily without difficulty	1
Yes the head of household can write with some difficulty	2
No the head of household cannot write at all	3
Don't know	99

2.2.15 Did the head of household attend school? [Also answer Yes if still attending school]

Yes	1	
No	2	→ 2.3.1
Don't Know	99	

2.2.16 [If Yes:] Until which grade/class did the head of household attend? circle

Primary	1	2	3	4	5	6	7	8
Secondary	1	2	3	4	5	6		
Tertiary	1	2	3	4	5			
Vocational	1	2	3	4				
Don't Know	99							

END OF QUESTIONS ABOUT HEAD OF HOUSEHOLD

2.3.1 How many other members of the household are presently working, besides the head of household and you? [ ][ ] if 00 → 2.3.9

What are the principal occupations of the other household members that are working?

[Write total number of persons working, for each occupation category]

2.3.2	Employed in private sector or by NGO	[ ][ ]
2.3.3	Civil servant	[ ][ ]
2.3.4	Casual worker (manual/agriculture)	[ ][ ]
2.3.5	Self-employed farmer/ peasant/fisherman/cattlekeeper	[ ][ ]
2.3.6	Self-employed business man/women	[ ][ ]
2.3.7	Domestic work in own household	[ ][ ]
2.3.8	Total number working:	[ ][ ]

[Compare answer to 2.3.8 with 2.3.1 Probe if total doesn't match 2.3.1]

2.3.9 What is the average monthly cash income from wages/salaries into your household?

[Include regular cash income sent to the household from family members elsewhere]

0.0 UGX / month	1
1 < 50,000 UGX/month	2
50,000 - <100.000 UGX/month	3
100,000 – <200,000 UGX/month	4
200,000 – <300,000 UGX/month	5
300,000 - <500,000 UGX/month	6
500,000 or more UGX/month	7
Don't Know	99

Does any member of the household own any of the following means of transport? [Read list and answer Yes/No]

2.3.10	an animal drawn cart	Yes	1	
		No	2	
2.3.11	a bicycle	Yes	1	
		No	2	
2.3.12	a motorcycle/scooter	Yes	1	
		No	2	
2.3.13	a car/truck	Yes	1	
		No	2	
2.3.14	a boat/canoe without motor	Yes	1	
		No	2	
2.3.15	a boat with motor	Yes	1	
		No	2	
2.3.16	Other	Yes	1	
		No	2	→ 2.3.18

2.3.17 **[If other:]** Specify ______________________________

2.3.18 How many rooms do you have in the household? Total number of rooms: ☐☐

2.3.19 How many rooms are used for sleeping? Rooms used for sleeping: ☐☐

2.3.20 What is the **MAIN** source of lighting for the household?

Kerosene/paraffin lantern	1	→ 2.3.22
Paraffin candle (Tadooba)	2	
Firewood	3	
Candle (Wax)	4	
Electricity	5	
Solar	6	
Dry Cell lamp	7	
Other	88	

2.3.21 **[If other:]** Specify ______________________________

Does your household have ANY of the following items in working condition? [Read out list and circle Yes/No]

2.3.22	electricity (fixed line)	Yes	1
		No	2
2.3.23	solar power/generator	Yes	1
		No	2
2.3.24	a radio	Yes	1
		No	2
2.3.25	a television	Yes	1
		No	2

2.3.26	a mobile phone	Yes 1<br>No 2	
2.3.27	a fixed phone	Yes 1<br>No 2	
2.3.28	a refrigerator	Yes 1<br>No 2	
2.3.29	a bed	Yes 1<br>No 2	
2.3.30	a foam mattress	Yes 1<br>No 2	
2.3.31	a sofa	Yes 1<br>No 2	
2.3.32	a cupboard (for clothing)	Yes 1<br>No 2	
2.3.33	a watch/clock	Yes 1<br>No 2	
2.3.34	a mosquito net that can be used while sleeping	Yes 1<br>No 2	→ 2.3.37
2.3.35	Did the youngest child sleep under a mosquito net last night?	Yes 1<br>No 2<br>Don't Know 99	
2.3.36	In total, how many of the children under the age of five slept under a mosquito net last night?	Don't Know=99 [ ][ ]	
2.3.37	Does any member of the household own any animals?	Yes 1<br>No 2	→ 2.3.49

[If Yes:] Does any member of the household own any of the following animals?
[Do not include Cats or Dogs]
[Fill how many of each, Fill 00000 if none. Fill 99999 if don't know]

2.3.38	Cows						
2.3.39	Horses, donkeys, or mules						
2.3.40	Goats						
2.3.41	Sheep						
2.3.42	Pigs						
2.3.43	Chickens or Ducks						
2.3.44	Turkeys						
2.3.45	Rabbits						
2.3.46	Beehives						
2.3.47	Other						0 → 2.3.49

2.3.48 **[If other:]** Specify ________________________________

form ID = ☐☐☐☐☐☐☐

2.3.49 Does any member of the household own land?

Yes	1
No	2 → 2.3.54

2.3.50 **[If Yes:]** Was it bought, inherited or given to a member of your household, or is your household "squatter"? **[Multiple answers possible]**

Bought	1
Inherited or given	2
Squatter	3

2.3.51 How much land do members of the household own?

☐☐☐ . ☐☐☐☐ Acres

Don't know 999.9999

[Use calculation if measurement is not given in acres. Write your calculation in full on this form and show your]supervisor for confirmation.]

2.3.52 How much of this land is agricultural land ?

☐☐☐ . ☐☐☐☐ Acres

Don't know 999.9999

2.3.53 How much of this land is for other purposes, like for a business or rented out?

☐☐☐ . ☐☐☐☐ Acres

Don't know 999.9999

2.3.54 What is the **MAIN** type of fuel used for cooking in the household?

Firewood	1 → 2.3.56
Charcoal	2
Paraffin/Kerosene	3
Gas	4
Electricity	5
Crop residue/Straw/Grass	6
Animal Dung	7
Solar	8
No food cooked in household	9
Other	88

2.3.55 **[If other:]** Specify ______________________________

2.3.56 **[Observe or ask:]** Where is the kitchen located?

In the main or only room	1
Separate room	2
Separate building	3
Outside	4

2.3.57 What is the **MAIN** source of drinking water for members of your household?

Piped water to home	1 → S3
Public tap	2
Unprotected well	3
Protected well	4
Unprotected spring	5
Protected spring	6
Borehole	7
River/stream	8
Stagnant pool/lake	9
Rainwater collection	10
Purchase water/truck	11
Other	88

2.3.58 **[If other:]** Specify ______________________________

S3 SECTION 3 : LIST ALL CHILDREN UNDER 5 AND THEIR DEMOGRAPHIC CHARACTERISTICS

[Re-confirm that the respondent is the primary caretaker of the children in this household.
Include all children under five, under the care of this household, who slept in this house last night.
Make sure the list isn't limited to the children of the caretaker only.]

[SAY:]
Now I will ask you some questions about the children under five years of age
Who are under your care in this household, and slept here last night.

3.1 How many children under age of 5 years, who slept here last night, are part of your household ? [ ][ ]

[Say:] **Now I will ask some questions about each of the children under five years of age**

CHILD 01

3.1.1 What is the child's name? ________________ **C 01**

3.1.2 Is the child less than one month old?

Yes	1
No	2

[If the child is less than one month old, write the age in WEEKS and DAYS. If the child is one month or older, write the age in YEARS and MONTHS]

3.1.3 What is the child's age? **WEEKS, DAYS** OR **YEARS, MONTHS** [ ] , [ ][ ]

3.1.4 What is the child's date of birth? <DD/MM/20YY> [ ][ ] / [ ][ ] / 2 0 [ ][ ]

dont know = 99/99/2099

3.1.5 What is the child's sex?

Male	1
Female	2

3.1.6 What is your relation to this child?

Mother	1	→ 3.2.1
Father	2	
Sibling	3	
Aunt/Uncle	4	
Grandfather/Grandmother	5	
Other	88	

3.1.7 **[If other:]** Specify ________________

CHILD 02

3.2.1 What is the child's name? ________________ **C 02**

3.2.2 Is the child less than one month old?

Yes	1
No	2

[If the child is less than one month old, write the age in WEEKS and DAYS. If the child is one month or older, write the age in YEARS and MONTHS]

3.2.3 What is the child's age? **WEEKS, DAYS** OR **YEARS, MONTHS** [ ] , [ ][ ]

3.2.4 What is the child's date of birth? <DD/MM/20YY> [ ][ ] / [ ][ ] / 2 0 [ ][ ]

dont know = 99/99/2099

3.2.5 What is the child's sex?

Male	1
Female	2

3.2.6 What is your relation to this child?

Mother	1	→ 3.3.1
Father	2	
Sibling	3	
Aunt/Uncle	4	
Grandfather/Grandmother	5	
Other	88	

3.2.7 **[If other:]** Specify ________________

form ID = ☐☐☐☐☐☐☐

CHILD 03

3.3.1 What is the child's name? ____________________ C 03

3.3.2 Is the child less than one month old?

Yes	1
No	2

[If the child is less than one month old, write the age in WEEKS and DAYS. If the child is one month or older, write the age in YEARS and MONTHS]

3.3.3 What is the child's age? **WEEKS, DAYS** OR **YEARS, MONTHS** ☐ , ☐☐

3.3.4 What is the child's date of birth? <DD/MM/20YY> ☐☐ / ☐☐ / 2 0 ☐☐

dont know = 99/99/2099

3.3.5 What is the child's sex?

Male	1
Female	2

3.3.6 What is your relation to this child?

Mother	1	→ 3.4.1
Father	2	
Sibling	3	
Aunt/Uncle	4	
Grandfather/Grandmother	5	
Other	88	

3.3.7 **[If other:]** Specify ____________________

CHILD 04

3.4.1 What is the child's name? ____________________ C 04

3.4.2 Is the child less than one month old?

Yes	1
No	2

[If the child is less than one month old, write the age in WEEKS and DAYS. If the child is one month or older, write the age in YEARS and MONTHS]

3.4.3 What is the child's age? **WEEKS, DAYS** OR **YEARS, MONTHS** ☐ , ☐☐

3.4.4 What is the child's date of birth? <DD/MM/20YY> ☐☐ / ☐☐ / 2 0 ☐☐

dont know = 99/99/2099

3.4.5 What is the child's sex?

Male	1
Female	2

3.4.6 What is your relation to this child?

Mother	1	→ 3.5.1
Father	2	
Sibling	3	
Aunt/Uncle	4	
Grandfather/Grandmother	5	
Other	88	

3.4.7 **[If other:]** Specify ____________________

CHILD 05

3.5.1 What is the child's name? ______________________ C 05

3.5.2 Is the child less than one month old?

Yes	1
No	2

[If the child is less than one month old, write the age in WEEKS and DAYS. If the child is one month or older, write the age in YEARS and MONTHS]

3.5.3 What is the child's age? **WEEKS, DAYS** OR **YEARS, MONTHS** [ ] , [ ][ ]

3.5.4 What is the child's date of birth? <DD/MM/20YY> [ ][ ] / [ ][ ] / 2 0 [ ][ ]

dont know = 99/99/2099

3.5.5 What is the child's sex?

Male	1
Female	2

3.5.6 What is your relation to this child?

Mother	1	→ 3.6.1
Father	2	
Sibling	3	
Aunt/Uncle	4	
Grandfather/Grandmother	5	
Other	88	

3.5.7 **[If other:]** Specify ______________________

CHILD 06

3.6.1 What is the child's name? ______________________ C 06

3.6.2 Is the child less than one month old?

Yes	1
No	2

[If the child is less than one month old, write the age in WEEKS and DAYS. If the child is one month or older, write the age in YEARS and MONTHS]

3.6.3 What is the child's age? **WEEKS, DAYS** OR **YEARS, MONTHS** [ ] , [ ][ ]

3.6.4 What is the child's date of birth? <DD/MM/20YY> [ ][ ] / [ ][ ] / 2 0 [ ][ ]

dont know = 99/99/2099

3.6.5 What is the child's sex?

Male	1
Female	2

3.6.6 What is your relation to this child?

Mother	1	→ 3.7.1
Father	2	
Sibling	3	
Aunt/Uncle	4	
Grandfather/Grandmother	5	
Other	88	

3.6.7 **[If other:]** Specify ______________________

form ID = ☐☐☐☐☐☐☐

CHILD 07

3.7.1 What is the child's name? ____________________ C 07

3.7.2 Is the child less than one month old?

Yes	1
No	2

[If the child is less than one month old, write the age in WEEKS and DAYS. If the child is one month or older, write the age in YEARS and MONTHS]

3.7.3 What is the child's age? **WEEKS, DAYS** OR **YEARS, MONTHS** ☐ , ☐☐

3.7.4 What is the child's date of birth? <DD/MM/20YY> ☐☐ / ☐☐ / 2 0 ☐☐

dont know = 99/99/2099

3.7.5 What is the child's sex?

Male	1
Female	2

3.7.6 What is your relation to this child?

Mother	1	→ 3.8.1
Father	2	
Sibling	3	
Aunt/Uncle	4	
Grandfather/Grandmother	5	
Other	88	

3.7.7 **[If other:]** Specify ____________________

CHILD 08

3.8.1 What is the child's name? ____________________ C 08

3.8.2 Is the child less than one month old?

Yes	1
No	2

[If the child is less than one month old, write the age in WEEKS and DAYS. If the child is one month or older, write the age in YEARS and MONTHS]

3.8.3 What is the child's age? **WEEKS, DAYS** OR **YEARS, MONTHS** ☐ , ☐☐

3.8.4 What is the child's date of birth? <DD/MM/20YY> ☐☐ / ☐☐ / 2 0 ☐☐

dont know = 99/99/2099

3.8.5 What is the child's sex?

Male	1
Female	2

3.8.6 What is your relation to this child?

Mother	1	→ 3.9.1
Father	2	
Sibling	3	
Aunt/Uncle	4	
Grandfather/Grandmother	5	
Other	88	

3.8.7 **[If other:]** Specify ____________________

CHILD 09

3.9.1 What is the child's name? ______________________ C 09

3.9.2 Is the child less than one month old?

Yes	1
No	2

[If the child is less than one month old, write the age in WEEKS and DAYS. If the child is one month or older, write the age in YEARS and MONTHS]

3.9.3 What is the child's age? **WEEKS, DAYS** OR **YEARS, MONTHS** [ ] , [ ][ ]

3.9.4 What is the child's date of birth? <DD/MM/20YY> [ ][ ] / [ ][ ] / 2 0 [ ][ ]

dont know = 99/99/2099

3.9.5 What is the child's sex?

Male	1
Female	2

3.9.6 What is your relation to this child?

Mother	1	→ 3.10.1
Father	2	
Sibling	3	
Aunt/Uncle	4	
Grandfather/Grandmother	5	
Other	88	

3.9.7 **[If other:]** Specify ______________________

CHILD 10

3.10.1 What is the child's name? ______________________ C 10

3.10.2 Is the child less than one month old?

Yes	1
No	2

[If the child is less than one month old, write the age in WEEKS and DAYS. If the child is one month or older, write the age in YEARS and MONTHS]

3.10.3 What is the child's age? **WEEKS, DAYS** OR **YEARS, MONTHS** [ ] , [ ][ ]

3.10.4 What is the child's date of birth? <DD/MM/20YY> [ ][ ] / [ ][ ] / 2 0 [ ][ ]

dont know = 99/99/2099

3.10.5 What is the child's sex?

Male	1
Female	2

3.10.6 What is your relation to this child?

Mother	1	→ 3.11
Father	2	
Sibling	3	
Aunt/Uncle	4	
Grandfather/Grandmother	5	
Other	88	

3.10.7 **[If other:]** Specify ______________________

form ID = ☐☐☐☐☐☐☐

[Say] Now I would like to ask whether any of these children have been sick in the last 2 weeks, including today:

3.11	In the last 2 weeks, including today, has <NAME> C01 been sick?	Yes 1<br>No 2
3.12	In the last 2 weeks, including today, has <NAME> C02 been sick?	Yes 1<br>No 2
3.13	In the last 2 weeks, including today, has <NAME> C03 been sick?	Yes 1<br>No 2
3.14	In the last 2 weeks, including today, has <NAME> C04 been sick?	Yes 1<br>No 2
3.15	In the last 2 weeks, including today, has <NAME> C05 been sick?	Yes 1<br>No 2
3.16	In the last 2 weeks, including today, has <NAME> C06 been sick?	Yes 1<br>No 2
3.17	In the last 2 weeks, including today, has <NAME> C07 been sick?	Yes 1<br>No 2
3.18	In the last 2 weeks, including today, has <NAME> C08 been sick?	Yes 1<br>No 2
3.19	In the last 2 weeks, including today, has <NAME> C09 been sick?	Yes 1<br>No 2
3.20	In the last 2 weeks, including today, has <NAME> C10 been sick?	Yes 1<br>No 2
3.21	Confirm total number of children sick in the past two weeks	☐☐<br>if 00 → S5

S4	SECTION 4. ILLNESS, CARE SEEKING AND TREATMENT

4.1.1 **[Copy the form id from the label on front page - section 1]** ☐☐☐☐☐☐☐

[Complete section 4 for one child's illness episode.]
[Then repeat on separate section 4 forms for the other sick children mentioned in section 3]

[For which child are you filling out this section 4?]
[Confirm with respondent from section 3:]

4.1.2 CHILD CODE= C ☐☐

4.1.3 NAME ____________________

4.1.4 What is the child's AGE **WEEKS, DAYS** OR **YEARS, MONTHS** ☐, ☐☐

	Question		
4.2.1	Has (name) had an illness with a cough at any time in the last 2 weeks?	Yes	1
		No	2 → 4.2.3
4.2.2	If Yes, when (name) had an illness with a cough, did he/she breathe faster than usual with short, rapid breaths?	Yes	1
		No	2
4.2.3	Has (name) been ill with a fever at any time in the last 2 weeks, including today?	Yes	1
		No	2
4.2.4	Has (name) been ill with diarrhoea at any time in the last 2 weeks, including today?	Yes	1
		No	2 → 4.2.7
4.2.5	How many stools in a day and the following night did (name) have?	☐☐	
4.2.6	Was the diarrhoea: **[probe]**	Watery	1
		Mucoid	2
		Bloody	3
4.2.7	Did (name) have any of the following symptoms: **[Read options]** Convulsions	Yes	1
		No	2
4.2.8	Unconsciousness	Yes	1
		No	2
4.2.9	Lethargy/abnormally sleepy	Yes	1
		No	2
4.2.10	Vomiting everything	Yes	1
		No	2
4.2.11	Not drinking/breastfeeding	Yes	1
		No	2
4.2.12	Chest indrawing	Yes	1
		No	2
4.3.1	Did you seek advice or treatment for the illness episode outside the home?	Yes	1 → 4.3.4
		No	2

form ID = ☐☐☐☐☐☐☐

4.3.2	**[If no:]** Why did you not seek advice or treatment outside the home? **[Circle MAIN reason]**	Health facility/ provider too far	1 → 4.5.1
		Had No time	2
		Had No money	3
		Did Not want to attend a health facility	4
		Could manage at home	5
		Spouse did Not allow	6
		Father's mother did Not allow	7
		Illness was Not severe	8
		Don't know	99
		Other (specify):	88
4.3.3	**[If other:]** Specify:	______________________	skip → 4.5.1
4.3.4	**[If yes:]** Where did you seek advice from? **[multiple answers possible]**	PUBLIC SECTOR:	
		Village health team/CMD	1 → 4.3.6
		Health centre II	2
		Health centre III	3
		Health centre IV	4
		Hospital	5
		Clinic/outreach services	6
		PRIVATE MEDICAL SECTOR:	
		Pvt. hospital/clinic	7
		Pharmacy/drug shop	8
		Pvt. doctor	9
		Clinic/outreach services	10
		OTHER SOURCE:	
		General Shop	11
		Traditional practitioner	12
		Other	88
4.3.5	**[If other:]** Specify:	______________________	

4.3.6 **[If only one alternative is circled above:] SKIP to** **4.3.9**

4.3.7 **[If two or more alternatives are circled above:]**
Where did you first seek advice or treatment?

PUBLIC SECTOR:		
Village health team/CMD	1	→ 4.3.9
Health centre II	2	
Health centre III	3	
Health centre IV	4	
Hospital	5	
Clinic/outreach services	6	
PRIVATE MEDICAL SECTOR:		
Pvt. hospital/clinic	7	
Pharmacy/drug shop	8	
Pvt. doctor	9	
Clinic/outreach services	10	
OTHER SOURCE:		
General Shop	11	
Traditional practitioner	12	
Other	88	

4.3.8 **[If other:]** Specify: ______________________

4.3.9 How did you get to the first place to seek advice or treatment from?
[Circle MAIN transport]

Walking	1	→ 4.3.11
Bicycling	2	
Boda Boda (motorbike taxi)	3	
Taxi (public minibus)	4	
Bus	5	
Special hire car	6	
Lorry	7	
Boat	8	
Other	88	

4.3.10 **[If other:]** Specify: text ______________________

4.3.11 How long did it take you to get from home to the first place you sought treatment/advice from? hrs: min [ ][ ] : [ ][ ]

4.3.12 In relation to this visit to the first place you sought advice or treatment from:
Did you spend any money on any of the following? **[Fill 0000,000 if no money was spent]**

4.3.13 Transportation UGX [ ][ ][ ][ ] , [ ][ ][ ]
Dont know = 9999,999

4.3.14 Medicines UGX [ ][ ][ ][ ] , [ ][ ][ ]
Dont know = 9999,999

4.3.15 Material costs (like gloves, iv, syringes etc) UGX [ ][ ][ ][ ] , [ ][ ][ ]
Dont know = 9999,999

4.3.16 Patient registration fees UGX [ ][ ][ ][ ] , [ ][ ][ ]
Dont know = 9999,999

4.3.17 Monetary gifts, contributions,gratitutes UGX [ ][ ][ ][ ] , [ ][ ][ ]
Dont know = 9999,999

form ID = ☐☐☐☐☐☐☐

4.3.18 Sustenance costs (e.g. Food, drinks) UGX ☐☐☐☐, ☐☐☐
Dont know = 9999,999

4.3.19 Other financial expenditure UGX ☐☐☐☐, ☐☐☐
Dont know = 9999,999

4.3.20 **[If other:]** Specify: text ______________________________

4.3.21 **[<u>CHECK</u> Q 4.3.4 : If only one place was mentioned:] SKIP to: 4.4.1**

[If two or more alternatives circled in Q 4.3.4]

4.3.22 What was the <u>second place</u> where you did seek advice or treatment?

PUBLIC SECTOR:		
Village health team/CMD	1	→ 4.3.24
Health centre II	2	
Health centre III	3	
Health centre IV	4	
Hospital	5	
Clinic/outreach services	6	
PRIVATE MEDICAL SECTOR:		
Pvt. hospital/clinic	7	
Pharmacy/drug shop	8	
Pvt. doctor	9	
Clinic/outreach services	10	
OTHER SOURCE:		
General Shop	11	
Traditional practitioner	12	
Other	88	

4.3.23 **[If other:]** Specify: text ______________________________

4.3.24 How did you get to the <u>second place</u> where you did seek advice or treatment from?
[Circle main transport]

Walking	1	→ 4.3.26
Bicycling	2	
Boda Boda (motorbike taxi)	3	
Taxi (public minibus)	4	
Bus	5	
Special hire car	6	
Lorry	7	
Boat	8	
Other	88	

4.3.25 **[If other:]** Specify: text ______________________________

4.3.26 How long did it take you to get from home to the <u>second place</u> you sought treatment/advice from? hrs: min ☐☐ : ☐☐

4.3.27 In relation to this visit to the <u>second place</u> you sought advice or treatment from:
Did you spend any money on any of the following? **[Fill 0000,000 if no money was spent]**

4.3.28 Transportation UGX ☐☐☐☐, ☐☐☐
don't know=9999,999

4.3.29 Medicines UGX [ ][ ][ ][ ] , [ ][ ][ ]

don't know=9999,999

4.3.30 Material costs (like gloves, iv, syringes etc) UGX [ ][ ][ ][ ] , [ ][ ][ ]

Dont know = 9999,999

4.3.31 Patient registration fees UGX [ ][ ][ ][ ] , [ ][ ][ ]

Dont know = 9999,999

4.3.32 Monetary gifts, contributions,gratitutes UGX [ ][ ][ ][ ] , [ ][ ][ ]

Dont know = 9999,999

4.3.33 Sustenance costs (e.g. Food, drinks) UGX [ ][ ][ ][ ] , [ ][ ][ ]

Dont know = 9999,999

4.3.34 Other financial spendlture UGX [ ][ ][ ][ ] , [ ][ ][ ]

Dont know = 9999,999 **if not 0000,000** → 4.4.1

4.3.35 **[If other:]** Specify: ______________________

4.4.1 Does this community have a VHT?

Yes	1	
No	2	→ 4.4.4
Don't know	99	

4.4.2 **[If yes, then CHECK answer to Q .3.4]**

[If yes, and the VHT was mentioned in Q 4.3.4 as a place where advice was sought from: SKIP to Q 4.4.4]

[If yes, and if the VHT was not mentioned in Q 4.3.4 as a place where advice was sought, then ask:]

Why did you not visit the Village Health Team ?

Don't know the VHT	1	→ 4.4.4
VHT Have No drugs	2	
VHT Too expensive	3	
VHT Too far	4	
VHT was Not available	5	
VHT Not good/ don't trust	6	
Too busy there	7	
Other:	88	

4.4.3 **[If other:]** Specify: ______________________

4.4.4 How many days after you first saw that the child was ill did you seek advice or treatment? DAYS [ ][ ]

4.4.5 Did anyone count the breathing of the child?

[Show respiratory rate timer]

Yes	1	
No	2	→ 4.4.8
Don't know	99	

form ID = ☐☐☐☐☐☐☐

4.4.6	**[If Yes:]** Who counted the breaths of the child?	Village health team/CMD	1	→ 4.4.8
		Staff in Health centre	2	
		Staff in Hospital	3	
		Staff at Private clinic	4	
		Staff at Pharmacy/drug shop	5	
		Other	88	
4.4.7	**[If other:]** Specify:	______________________		
4.4.8	At any time during the illness, did (name) have blood taken from his/her finger or heel for testing?	Yes	1	
		No	2	→ 4.5.1
		Don't know	99	
4.4.9	**[If yes:]** By whom?	Village health team/CMD	1	→ 4.4.11
		Staff in Health centre	2	
		Staff in Hospital	3	
		Staff at Private clinic	4	
		Staff at Pharmacy/drug shop	5	
		Other	88	
4.4.10	**[If other:]** Specify:	______________________		
4.4.11	Was this test an RDT, like this? **[Show RDT]**	Yes	1	
		No	2	
		Don't know	99	
4.4.12	Was the test positive for malaria?	Yes	1	
		No	2	
		Don't know	99	

4.5.1	At any time during the illness episode, did (name) take any drugs for the illness?	Yes	1	
		No	2	→ 4.7.4
		Don't know	99	

If yes, we want to find out which drugs were taken.
Probe in this order:
1. Ask to see balance
2. Ask to see treatment card / prescription
3. Then show the drug posters one by one.

4.5.2	**[Show examples of drugs on card 1 and ask]** Did (name) receive any of these drugs? **[Let respondent identify any drugs on this poster]**	Yes	1	
		No	2	→ 4.5.4
		Don't know	99	

4.5.3 **[If yes, circle the code(s):]** circle

D1	D2	D3	D4	D5	D6

4.5.4 **[Answer for each of the drugs mentioned below if the respondent has either shown the drug, or a prescription, or identified the drug on the poster. If so, circle YES. Otherwise circle NO. Do not probe]**

4.5.5 **ORS (D2)**

Yes	1
No	2

4.5.6 **ZINC (D3)**

Yes	1
No	2

4.5.7 **[If the respondent mentioned spontaneously that they used home made ORS, circle Yes for Home-made ORS. Do not probe.]**

Homemade ORS

Yes	1
No	2

4.5.8 **[Show examples of drugs on card 2 and ask:]**

Did (name) receive any of these drugs?

[Let respondent identify any drugs on this poster]

Yes	1	
No	2	→ 4.5.10
Don't know	99	

4.5.9 **[If yes, circle the code(s):]** circle

P1	P2	P3	P4	P5	P6	P7	P8	P9

4.5.10 **[Answer for each of the drugs mentioned below if the respondent has either shown the drug, or a prescription, or identified the drug on the poster. If so, circle YES. Otherwise circle NO. Do not probe]**

4.5.11 **Amoxycillin (P1 or P2)**

Yes	1
No	2

4.5.12 **Ampicillin (P3)**

Yes	1	
No	2	→ 4.5.15

4.5.13 ***if yes , Ampicillin was taken,*** where did you get it from?

Village health team/CMD	1	→ 4.5.15
Health centre	2	
Hospital	3	
Private clinic	4	
Pharmacy/drug shop	5	
Other	88	

4.5.14 **[If other:]** Specify: ______________________

4.5.15 **Cotrimoxazole or Septrin (P4)**

Yes	1	
No	2	→ 4.5.18

4.5.16 ***if yes , Cotrimoxazole or Septrin was taken,*** where did you get it from?

Village health team/CMD	1	→ 4.5.18
Health centre	2	
Hospital	3	
Private clinic	4	
Pharmacy/drug shop	5	
Other	88	

4.5.17 **[If other:]** Specify: ______________________

form ID = ☐☐☐☐☐☐☐

4.5.18	**Chloramphenicol (P5)**	Yes	1	
		No	2	→ 4.5.21
4.5.19	***If yes , chloramphenicol was taken,*** where did you get it from?	Village health team/CMD	1	→ 4.5.21
		Health centre	2	
		Hospital	3	
		Private clinic	4	
		Pharmacy/drug shop	5	
		Other	88	
4.5.20	**[If other:]** Specify:	____________________		
4.5.21	**Erythromycin (P6)**	Yes	1	
		No	2	→ 4.5.24
4.5.22	***If yes , erythromycin was taken,*** where did you get it from?	Village health team/CMD	1	→ 4.5.24
		Health centre	2	
		Hospital	3	
		Private clinic	4	
		Pharmacy/drug shop	5	
		Other	88	
4.5.23	**[If other:]** Specify:	____________________		
4.5.24	**Azythromycin (P7)**	Yes	1	
		No	2	→ 4.5.27
4.5.25	***if yes , Azythromycin was taken,*** where did you get it from?	Village health team/CMD	1	→ 4.5.27
		Health centre	2	
		Hospital	3	
		Private clinic	4	
		Pharmacy/drug shop	5	
		Other	88	
4.5.26	**[If other:]** Specify:	____________________		
4.5.27	**Pen V (P8)**	Yes	1	
		No	2	→ 4.5.30
4.5.28	***if yes , Pen V was taken,*** where did you get it from?	Village health team/CMD	1	→ 4.5.30
		Health centre	2	
		Hospital	3	
		Private clinic	4	
		Pharmacy/drug shop	5	
		Other	88	
4.5.29	**[If other:]** Specify:	____________________		

4.5.30 **Cephalexin (P9)**

Yes	1		
No	2	→ 4.5.33	

4.5.31 ***if yes , Cephalexin was taken,*** where did you get it from?

Village health team/CMD	1	→ 4.5.33
Health centre	2	
Hospital	3	
Private clinic	4	
Pharmacy/drug shop	5	
Other	88	

4.5.32 **[If other:]** Specify: ____________________

4.5.33 **[Show examples of drugs on card 3 and ask:]**

Did (name) receive any of these drugs?

[Let respondent identify any drugs on this poster]

Yes	1	
No	2	→ 4.5.35
Don’t know	99	

4.5.34 **[If yes, circle the code(s):]** circle

M1	M2	M3	M4	M5
M6	M7	M8	M9	M10

[Answer for each of the drugs mentioned below if the respondent has either shown the drug, or a prescription, or identified the drug on the poster. If so, circle YES. Otherwise circle NO. Do not probe]

4.5.35 **Coartem**, Artemether-Lumefantrine (M1)

Yes	1
No	2

4.5.36 **Duo-Cotecxin (M2)**

Yes	1	
No	2	→ 4.5.39

4.5.37 ***if yes , Duo-Cotecxin was taken,*** where did you get it from?

Village health team/CMD	1	→ 4.5.39
Health centre	2	
Hospital	3	
Private clinic	4	
Pharmacy/drug shop	5	
Other	88	

4.5.38 **[If other:]** Specify: ____________________

form ID = ☐☐☐☐☐☐☐

4.5.39	**Rectal Artesunate/ Artenam** (M3)	Yes	1
		No	2 → 4.5.42
4.5.40	***if yes , Rectal Artesunate/ Artenam was taken,*** where did you get it from?	Village health team/CMD	1 → 4.5.42
		Health centre	2
		Hospital	3
		Private clinic	4
		Pharmacy/drug shop	5
		Other	88
4.5.41	**[If other:]** Specify: ______________________		
4.5.42	**Artemether (M4)**	Yes	1
		No	2 → 4.5.45
4.5.43	***if yes , Artemether was taken,*** where did you get it from?	Village health team/CMD	1 → 4.5.45
		Health centre	2
		Hospital	3
		Private clinic	4
		Pharmacy/drug shop	5
		Other	88
4.5.44	**[If other:]** Specify: ______________________		
4.5.45	**Fansidar or SP (M5)**	Yes	1
		No	2 → 4.5.48
4.5.46	***if yes , Fansidar or SP was taken,*** where did you get it from?	Village health team/CMD	1 → 4.5.48
		Health centre	2
		Hospital	3
		Private clinic	4
		Pharmacy/drug shop	5
		Other	88
4.5.47	**[If other:]** Specify: ______________________		
4.5.48	**Amodiaquine (M6)**	Yes	1
		No	2 → 4.5.51
4.5.49	***if yes , Amodiaquine was taken,*** where did you get it from?	Village health team/CMD	1 → 4.5.51
		Health centre	2
		Hospital	3
		Private clinic	4
		Pharmacy/drug shop	5
		Other	88
4.5.50	**[If other:]** Specify: ______________________		

4.5.51	**Amodiaquin - Artesunate (or Artesunate-Amodiaquine) (M7)**	Yes	1	
		No	2	→ 4.5.54
4.5.52	***if yes , Artesunate -Amodiaquine was taken,*** where did you get it from?	Village health team/CMD	1	→ 4.5.54
		Health centre	2	
		Hospital	3	
		Private clinic	4	
		Pharmacy/drug shop	5	
		Other	88	
4.5.53	**[If other:]** Specify:	___________________		
4.5.54	**Quinine (M8)**	Yes	1	
		No	2	→ 4.5.57
4.5.55	***if yes , Quinine was taken*** , where did you get it from?	Village health team/CMD	1	→ 4.5.57
		Health centre	2	
		Hospital	3	
		Private clinic	4	
		Pharmacy/drug shop	5	
		Other	88	
4.5.56	**[If other:]** Specify:	___________________		
4.5.57	**Chloroquine (M9)**	Yes	1	
		No	2	→ 4.5.60
4.5.58	***if yes , Chloroquine was taken,*** where did you get it from?	Village health team/CMD	1	→ 4.5.60
		Health centre	2	
		Hospital	3	
		Private clinic	4	
		Pharmacy/drug shop	5	
		Other	88	
4.5.59	**[If other:]** Specify:	___________________		
4.5.60	**Artesunate (M10)**	Yes	1	
		No	2	→ 4.5.63
4.5.61	***if yes , Artesunate -Fansidar was taken,*** where did you get it from?	Village health team/CMD	1	→ 4.5.63
		Health centre	2	
		Hospital	3	
		Private clinic	4	
		Pharmacy/drug shop	5	
		Other	88	
4.5.62	**[If other:]** Specify:	___________________		

form ID = ☐☐☐☐☐☐☐

4.5.63	**Artesunate -Fansidar**	Yes	1
		No	2 → 4.6.1
4.5.64	***if yes , Artesunate -Fansidar was taken,*** where did you get it from?	Village health team/CMD	1 → 4.6.1
		Health centre	2
		Hospital	3
		Private clinic	4
		Pharmacy/drug shop	5
		Other	88
4.5.65	**[If other:]** Specify: ________________________________		

	**[<u>CHECK</u> answer to Q 4.5.5. Was ORS one of the drugs taken?]**		
4.6.1	Where did you get the **ORS** from ?	Not applicable, did Not take ORS	77 → 4.6.7
		Village health team/CMD	1 → 4.6.3
		Health centre II	2
		Health centre III	3
		Health centre IV	4
		Hospital	5
		Clinic/outreach services	6
		PRIVATE MEDICAL SECTOR:	
		Pvt. hospital/clinic	7
		Pharmacy/drug shop	8
		Pvt. doctor	9
		Clinic/outreach services	10
		OTHER SOURCE:	
		General Shop	11
		Traditional practitioner	12
		Other	88
4.6.2	**[If other:]** Specify: ________________________		
4.6.3	How many days after you first saw that (name) was ill did (name) first take ORS?	Same day	0
		One day after	1
		2 days after	2
		Three or more days after	3
		Don't know	99

4.6.4	For how many days did (name) take **ORS?**		☐
4.6.5	What dose of **ORS** was (name) prescribed to take ?	1 sachet in 2 cups of water	1 → 4.6.7
		1 sachet in 1 cup of water	2
		Other	88
		Don't know	99 → 4.6.7
4.6.6	**[If other:]** Specify: ______________________________		
4.6.7	**[<u>CHECK</u> answer to Q 4.5.6. Was ZINC one of the drugs taken?]** Where did you get the **ZINC** from ?	Not applicable, did Not take ZINC	77 → 4.6.15
		Village health team/CMD	1 → 4.6.9
		Health centre II	2
		Health centre III	3
		Health centre IV	4
		Hospital	5
		Clinic/outreach services	6
		PRIVATE MEDICAL SECTOR:	
		Pvt. hospital/clinic	7
		Pharmacy/drug shop	8
		Pvt. doctor	9
		Clinic/outreach services	10
		OTHER SOURCE:	
		General Shop	11
		Traditional practitioner	12
		Other	88
4.6.8	**[If other:]** Specify: ______________________________		
4.6.9	How many days after you first saw that (name) was ill did (name) first take ZINC?	Same day	0
		One day after	1
		2 days after	2
		Three or more days after	3
		Don't know	99
4.6.10	For how many days did (name) take **ZINC?** number		☐

form ID = ☐☐☐☐☐☐☐

4.6.11 How many times a day did (name) take **ZINC?**

Once a day	1	→ 4.6.13
Two Times a day	2	
Three Times a Day	3	
More than three times a day	4	
Other	88	
Don't know	99	→ 4.6.13

4.6.12 **[If other:]** Specify: ____________________

4.6.13 What dose of **ZINC** was (name) prescribed to take ?

Half a tablet	1	→ 4.6.15
One tablet	2	
More than 1 tablet	3	
Other	88	
Don't know	99	→ 4.6.15

4.6.14 **[If other:]** Specify: ____________________

[<u>CHECK</u> answer to Q 4.5.11. Was AMOXYCILLIN one of the drugs taken?]

4.6.15 Where did you get the **AMOXYCILLIN** from ?

Not applicable, did Not take AMOXYCILLIN	77	→ 4.6.23
Village health team/CMD	1	→ 4.6.17
Health centre II	2	
Health centre III	3	
Health centre IV	4	
Hospital	5	
Clinic/outreach services	6	
PRIVATE MEDICAL SECTOR:		
Pvt. hospital/clinic	7	
Pharmacy/drug shop	8	
Pvt. doctor	9	
Clinic/outreach services	10	
OTHER SOURCE:		
General Shop	11	
Traditional practitioner	12	
Other	88	

4.6.16 **[If other:]** Specify: ____________________

4.6.17 How many days after you first saw that (name) was ill did (name) first take: **AMOXYCILLIN?**

Same day	0
One day after	1
2 days after	2
Three or more days after	3
Don't know	99

4.6.18 For how many days did (name) take **AMOXYCILLIN?** ☐

4.6.19 How many times a day did (name) take **AMOXYCILLIN?**

Once a day	1	→ 4.6.21
Two Times a day	2	
Three Times a Day	3	
More than three times a day	4	
Other	88	
Don't know	99	→ 4.6.21

4.6.20 **[If other:]** Specify: ____________________________

4.6.21 What dose of **AMOXYCILLIN** was (name) prescribed to take ?

1 tablet or capsule	1	→ 4.6.23
2 tablets or capsules	2	
More than 2 tablets or capsules	3	
Other	88	
Don't know	99	→ 4.6.23

4.6.22 **[If other:]** Specify: ____________________________

[<u>CHECK</u> answer to Q 4.5.35. Was COARTEM one of the drugs taken?]

4.6.23 Where did you get the **Coartem** from ?

Not applicable, did Not take COARTEM	77	→ 4.7.1
Village health team/CMD	1	→ 4.6.25
Health centre II	2	
Health centre III	3	
Health centre IV	4	
Hospital	5	
Clinic/outreach services	6	
PRIVATE MEDICAL SECTOR:		
Pvt. hospital/clinic	7	
Pharmacy/drug shop	8	
Pvt. doctor	9	
Clinic/outreach services	10	
OTHER SOURCE:		
General Shop	11	
Traditional practitioner	12	
Other	88	

4.6.24 **[If other:]** Specify: ____________________________

4.6.25 How many days after you first saw that (name) was ill did (name) first take **COARTEM?**

Same day	0
One day after	1
2 days after	2
Three or more days after	3
Don't know	99

4.6.26 For how many days did (name) take **COARTEM?** ☐

form ID = ☐☐☐☐☐☐☐

4.6.27	How many times a day did (name) take **COARTEM?**	Once a day	1	→ 4.6.29
		Two Times a day	2	
		Three Times a Day	3	
		More than three times a day	4	
		Other	88	
		Don't know	99	→ 4.6.29
4.6.28	**[If other:]** Specify:	___________________________		
4.6.29	What dose of **COARTEM** was (name) prescribed to take ?	Half a tablet	1	→ 4.7.1
		One tablet	2	
		Two tablets	3	
		More than 2 tablets	4	
		Other,	88	
		Don't know	99	→ 4.7.1
4.6.30	**[If other:]** Specify:	___________________________		
4.7.1	**[Ask for any of the drugs given:]** Did you give the treatment according to the instructions you were given (in terms of doses and frequency and number of days) by the health provider/ VHT?	Yes	1	→ 4.7.4
		No	2	
		Don't know	99	→ 4.7.4
4.7.2	If no, why did you not give the treatment according to the instructions that you were given by the health provider/VHT?	The child vomited	1	→ 4.7.4
		The medicine had a bad taste	2	
		The child developed a rash	3	
		The child developed diarrhoea	4	
		The child got cured	5	
		The child did Not improve	6	
		I forgot	7	
		Duration of treatment was too long	8	
		Other	88	
4.7.3	**[If other:]** Specify:	________________________		
4.7.4	[CHECK with section 3 whether there are more children who were sick in the past two weeks.]	Yes	1	→ 4.7.5
		No	2	→ 4.7.6

4.7.5 [If yes: fill out the "ADDITIONAL CHILD FORM" now]

4.7.6 [If no: continue to section 5.]

S5 SECTION 5. QUESTIONS ABOUT AWARENESS OF ZINC AND AMOXYCILLIN

Questions regarding **ZINC**
[**CHECK** answer to question........... **4.5.6**]

5.1 **[Don't ask but fill:]** Was **ZINC** taken?

Yes	1 → 5.4
No	2

5.2 Have you heard of a product called **ZINC**?

Yes	1
No	2 → 5.9
Don't know	99

5.3 **[If yes, the respondent has either mentioned ZINC before, or has heard of ZINC, ask:]**

Do you agree or disagree with the following statements:

5.4 **ZINC** is an effective treatment for diarrhoea.

Strongly agree	1
Somewhat agree	2
Somewhat disagree	3
Strongly disagree	4
Don't know	99

5.5 **ZINC** strengthens the child.

Strongly agree	1
Somewhat agree	2
Somewhat disagree	3
Strongly disagree	4
Don't know	99

5.6 **ZINC** should be taken with ORS.

Strongly agree	1
Somewhat agree	2
Somewhat disagree	3
Strongly disagree	4
Don't know	99

5.7 **ZINC** prevents diarrhoea.

Strongly agree	1
Somewhat agree	2
Somewhat disagree	3
Strongly disagree	4
Don't know	99

5.8 **ZINC** reduces diarrhoea.

Strongly agree	1
Somewhat agree	2
Somewhat disagree	3
Strongly disagree	4
Don't know	99

Questions regarding AMOXYCILLIN
[**CHECK** answer to question: .... **4.5.11**]

5.9	**[Don't ask but fill:]** Was **AMOXYCILLIN** taken?	Yes	1 → 5.11
		No	2
5.10	Have you heard of a product called **AMOXYCILLIN**?	Yes	1
		No	2 → S6
		Don't know	99

5.11 **[If yes, the respondent has either mentioned AMOXYCILLIN before, or has heard of AMOXYCILLIN, ask:]**

5.12	Would you give **AMOXYCILLIN** to your child if the child had:	Cough and a stuffy nose without fever or fast breathing?	Yes	1
			No	2
			Don't know	99
513		Cough and a stuffy nose and fever?	Yes	1
			No	2
			Don't know	99
5.14		Cough and fast breathing?	Yes	1
			No	2
			Don't know	99
5.15		In any other situation?	Yes	1
			No	2 → 5.17
5.16	**[If other:]** Specify	________________________________		

5.17 Do you agree or disagree with the following statement about **AMOXYCILLIN:**

**Amoxycillin** is an effective treatment for pneumonia.	Strongly agree	1
	Somewhat agree	2
	Somewhat disagree	3
	Strongly disagree	4
	Don’t know	99

S6 SECTION 6: VHT VISITS AND PREVENTIVE HEALTH BEHAVIORS

[CHECK section 4, Q 4.4.1: If the respondent did report any sick children in the past two weeks, then you have already asked question 6.1 , JUST FILL 6.1 and then continue to Q 6.2]

6.1 Does this community have a VHT?
Note **VHT= "the ones who give out drugs for children"**

Yes	1	
No	2	→ 6.4
Don't know	99	

6.2 If yes, Was your household visited by a VHT in the last month?

Yes	1	
No	2	→ 6.4
Don't know	99	

6.3 What did the VHT do during this visit?

Ask about the vaccinations of my child/children	Yes	1
	No	2
Kept record of health in my family	Yes	1
	No	2
Gave advice about healthy habits	Yes	1
	No	2
Asked or Observed health behaviour, practices,hygiene, and health condition of household members	Yes	1
	No	2

6.4 **[CHECK with section 3: Are there any children under age of 2 months:]**

Yes	1	
No	2	→ S7

[Continue this section only if you speak , or can speak, to the MOTHER of the child. If you can not, then SKIP to: S 7

6.5 Did you see anyone for antenatal care during this pregnancy?

Yes	1	
No	2	→ 6.8
Don't know	99	

6.6 How many months pregnant were you when you first received antenatal care for this pregnancy? DON'T Know =99 [ ][ ]

6.7 How many times did you receive antenatal care during this pregnancy? DON'T Know =99 [ ][ ]

6.8 Where did you deliver your baby?

At home	1	→ 6.9
At the TBA's home	2	
Health centre	3	
Private clinic	4	
Hospital	5	
At a midwife's /maternity home	6	
Other	88	

6.8.1 **[If other:]** Specify ________________________________

6.9 **[CHECK Q 6.1 : If there is no VHT in this village : SKIP to** **6.14**

6.10 Were you visited by a VHT in the first week of (name)'s life?

Yes	1	
No	2	→ 6.14
Don't know	99	

form ID = ☐☐☐☐☐☐☐

6.11	How many times were you visited in the first week ?	DON'T Know =99	☐☐
6.12	How long after delivery did the VHT first visit you?	On day of birth	1
		On the second day	2
		On the third day	3
		On the fourth day	4
		On the fifth day	5
		On the sixth	6
		On the seventh day	7
6.13	Did the VHT look at (name)'s health?	Yes	1
		No	2
		Don't know	99
6.14	**[SAY:] Now I would like to ask you some questions about how (name) was taken care of right after it was born.**		
6.15	Was anything ever applied to (name)'s cord?	Yes	1
		No	2 → 6.18
		Don't know	99
6.16	**[If Yes:]** What was applied to the cord?	Medical drugs	1 → 6.18
		Baby Powder	2
		Ash	3
		Salty water	4
		Cow dung	5
		Spirit	6
		Herbs	7
		Other	88
6.17	**[If other:]** Specify	________________	
6.18	Was (name) wiped (dried) as soon as she/he was born?	Yes	1
		No	2 → 6.20
		Don't know	99
6.19	[If Yes:] How soon after birth was (name) wiped (dried)?	Baby was not wiped/dried at all	1
		Within 15 minutes after birth	2
		After 15 but within 30 minutes	3
		After 30 but within 60 minutes	4
		Between 1 and 3 hours	5
		After three hours	6
6.20	How long after birth did you first put (name) to the breast?	Baby is Not breast fed at all	1
		Within one hour	2
		After one hour, but within three hours	3
		After three hours but within 12 hours	4
		After 12 but within 24 hours	5
		After 24 hours	6
		Don't know	99

S7 **Section 7 Question on MEDIA**

Regardless of previous answers about VHTS, in this section we want to know whether respondent has heard about VHTS, and if yes, how?

7 Ask: **SINCE NEW YEAR have you**:

7.1	Heard about VHTs on radio?	Yes	1
		No	2
7.2	Seen anything about VHTs on television?	Yes	1
		No	2
7.3	Read about VHTs in a newspaper or magazine?	Yes	1
		No	2
7.4	Heard about a VHTs by word of mouth?	Yes	1
		No	2

This was the last question in the questionnaire. SAY

“ This is now almost the end of the interview"

"Before I leave you, I would like to make some observations of this compound. This is also part of our study"

“Thank you very much for taking part in this survey. Do you have any questions you wanted to ask me?”

form ID = ☐☐☐☐☐☐☐

S8 Section 8. COMPOUND OBSERVATION

The following questions need to be answered at the end of the entire interview before you leave for the next household

	Question			
8.1	**[Observe:]** Can you easily see a washing basin/bowl/jerry can?	Yes	1	
		No	2	
8.2	**[Observe:]** Can you easily see soap for handwashing?	Yes	1	
		No	2	
8.3	**[Observe or Ask:]** What kind of toilet facility do members of your household usually use?	Flush toilet (sink tank)	1	→ 8.5
		VIP latrine	2	
		Ecosan	3	
		Pit latrine	4	
		Shared/public toilet	5	
		No facility(/bush/field)	6	
		Other	88	
8.4	**[If other:]** Specify ______________________________			
8.5	**[Observe:]** What is the **MAIN** material the roof is made of? **[Majority of roof]**	Thatch	1	→ 8.7
		Mud/clay	2	
		Grass/papyrus/banana leaf	3	
		**FINISHED:**		
		Wood planks	4	
		Iron/zinc/aluminium sheets	5	
		Asbestos	6	
		Tiles	7	
		Tin	8	
		Cement	9	
		Other	88	
8.6	**[If other:]** Specify ______________________________________			

8.7 **[Observe and record:]** What is the **MAIN** material the walls are made of?

[Majority of walls]

**NATURAL:**		
Reed	1	→ 8.9
Grass	2	
**RUDIMENTARY:**		
Mud	3	
unburned Bricks	4	
**FINISHED:**		
burned bricks	5	
Unplastered cement	6	
Plastered cement	7	
Stone	8	
Wood	9	
Other	88	

8.8 **[If other:]** Specify ______________________________

8.9 **[Observe and record:]** What is the material the window frames are made of?

[Majority of windows]

Reed	1	→ 8.11
Wood	2	
Open space wihout curtains	3	
Open space with curtains	4	
Aluminium/steel	5	
No windows	6	
Other	88	

8.10 **[If other:]** Specify ______________________________

8.11 **[Observe and record:]** What is the **MAIN** material the floors are made of?

**NATURAL:**		
Sand/gravel	1	→ 8.13
Earth	2	
Earth and cowdung	3	
**FINISHED:**		
Wood / Planks	4	
Cement	5	
Other	88	

8.12 **[If other:]** Specify ______________________________

[Use your GPS to identify the coordinates of this compound:]

8.13 Coordinates of compound NORTH [ ][ ]° [ ][ ] [ ][ ][ ]**'' N**

8.14 Coordinates of compound EAST [ ][ ][ ]° [ ][ ] [ ][ ][ ]**'' E**

exit **This is the end of your work with this household**

Thank the family again before moving to the next household
