# Supplementary material for: Variation in the quality and out-of-pocket cost of treatment for childhood malaria, diarrhoea, and pneumonia: Community and facility based care in rural Uganda
Source: PLoS One. 2018 Nov 26;13(11):e0200543. doi: 10.1371/journal.pone.0200543 (PMC6261061; doi:10.1371/journal.pone.0200543)

CARD 1 1. Supporting Information Figure 1. S1 Figure. inSCALE Cross-sectional baseline survey Drug Picture Cards.

Metronidazole/Flagyl (D1)

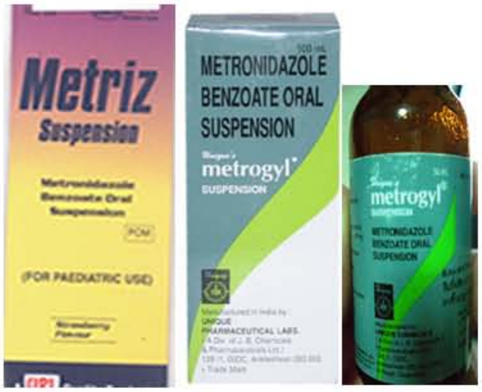

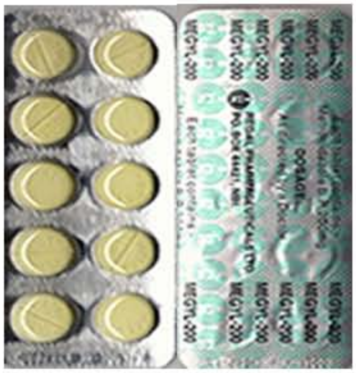

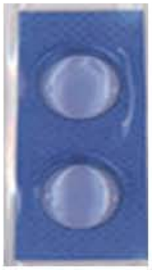

ORS (D2)

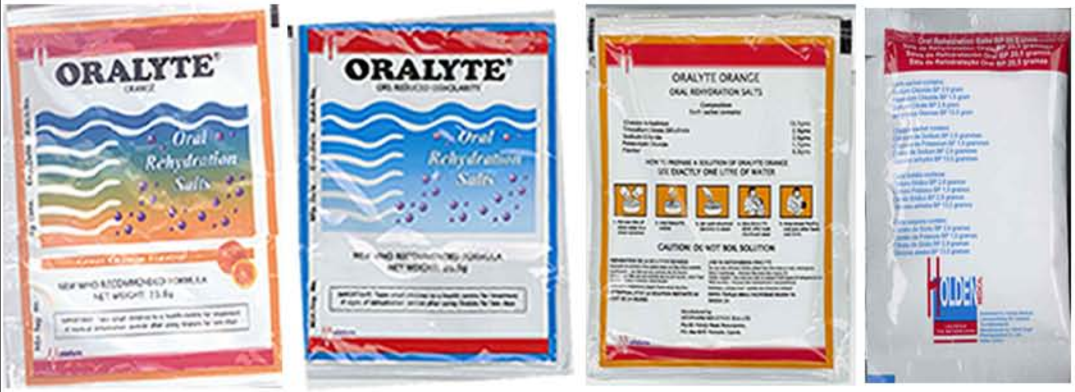

ZINC (D3)

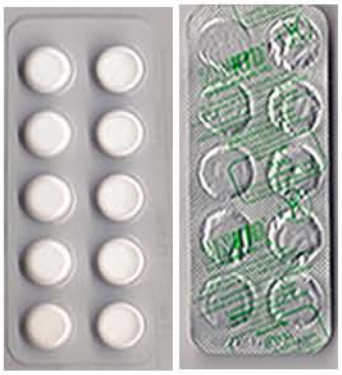

Deworming tablets

Levamisole (D4)

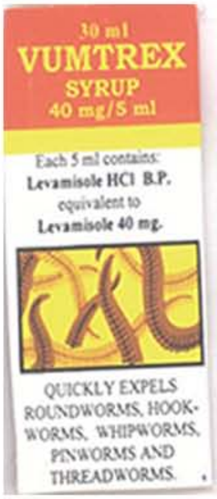

Albendazole (D5)

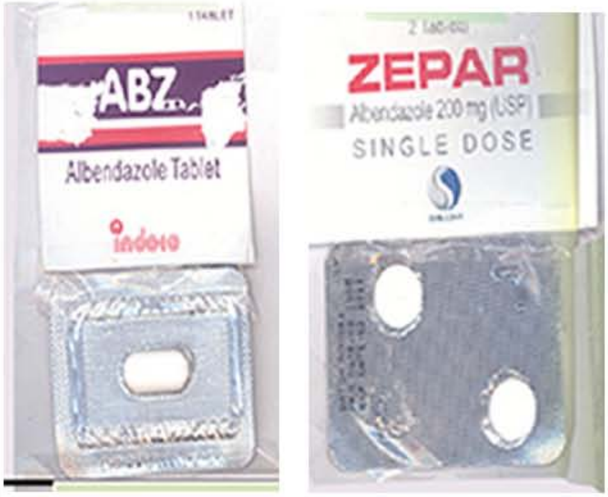

Mebandazole (D6)

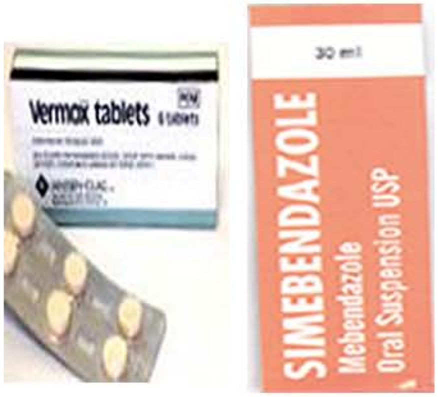

CARD 2

Amoxycillin (P1)

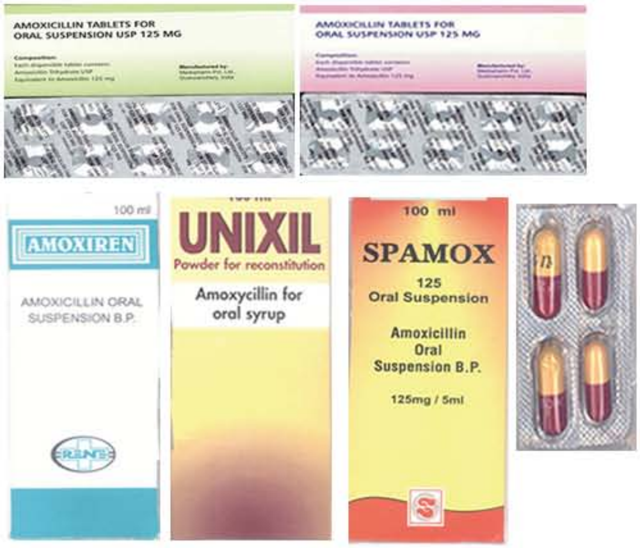

Co-Amoxycillin (P2)

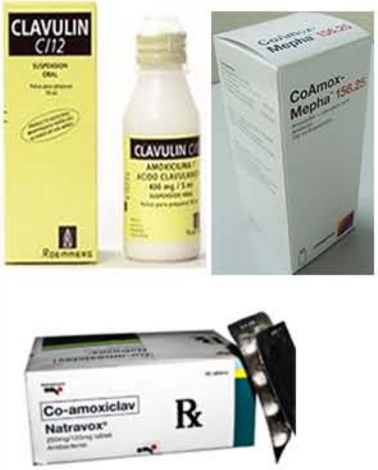

Ampicillin (P3)

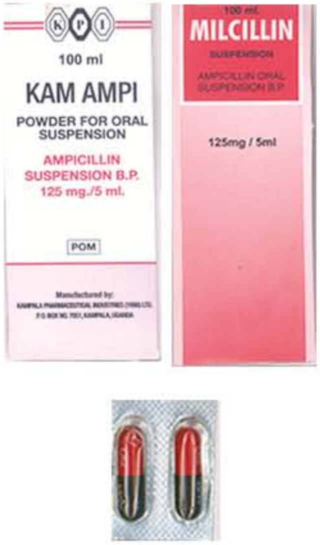

Cotrimoxazole/Septrin (P4)

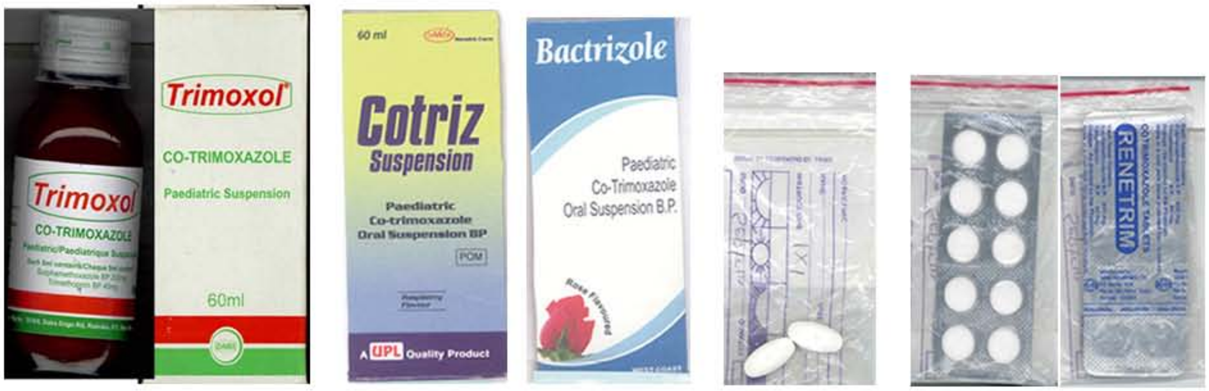

Chloramphenicol (P5)

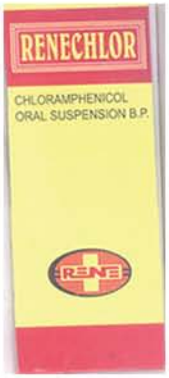

Erythromycin (P6)

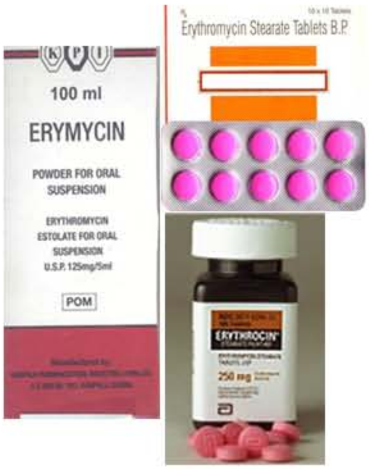

Azithromycin (P7)

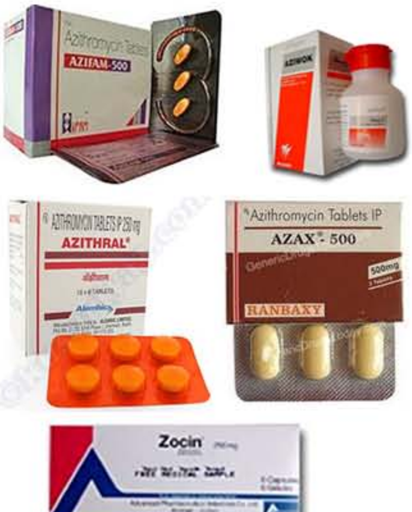

PEN V (P8)

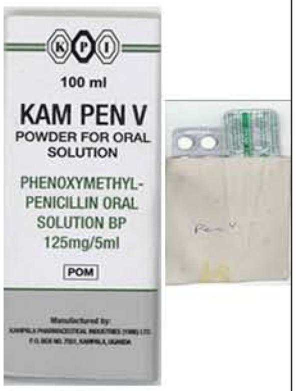

Cephalexin (P9)

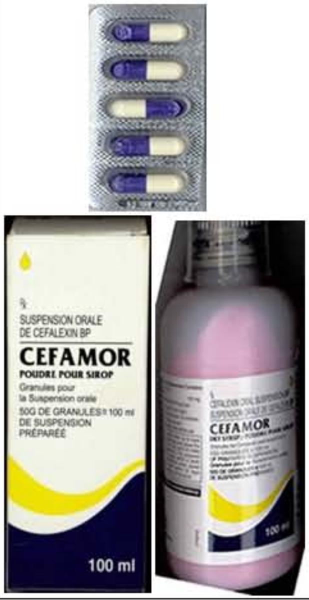

CARD 3

Coartem(M1)

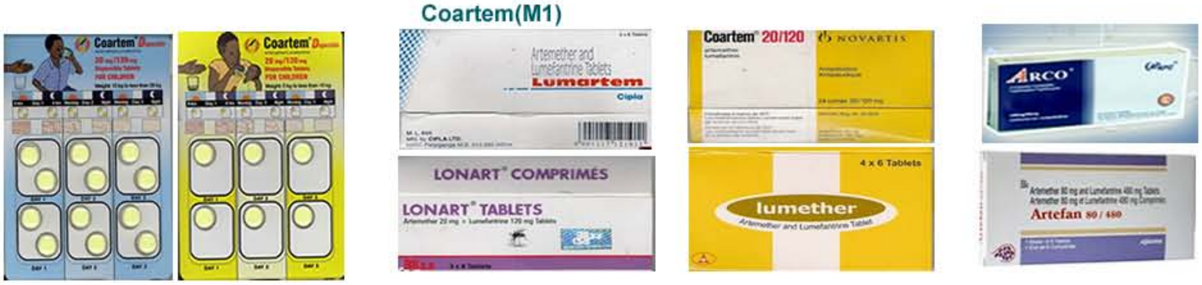

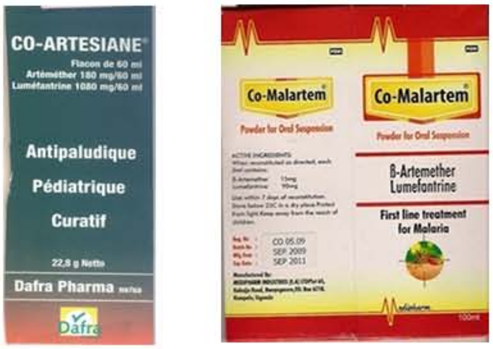

Duo-Cotecxin(M2)

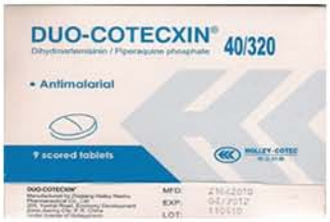

Rectal artesunate (M3)

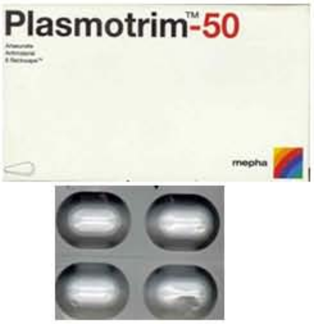

Artemether (M4)

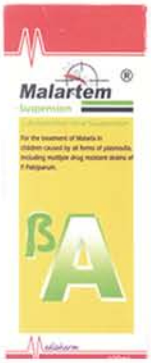

Fasindar/ SP (M5)

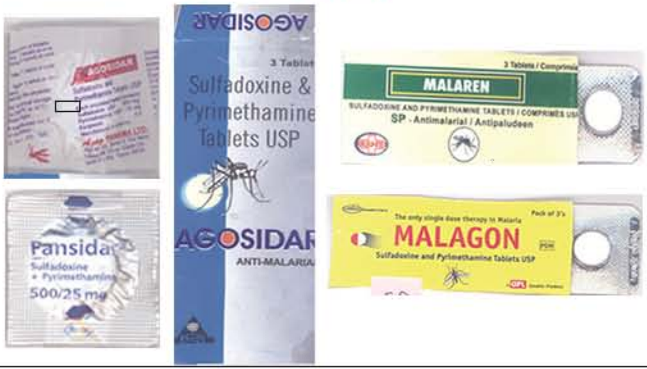

Amodiaquine(M6)

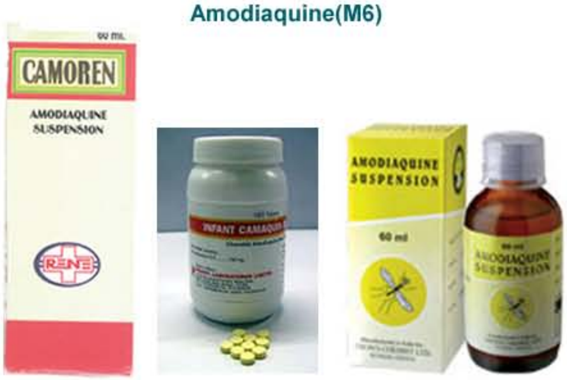

Amodiaquine/artesunate (M7)

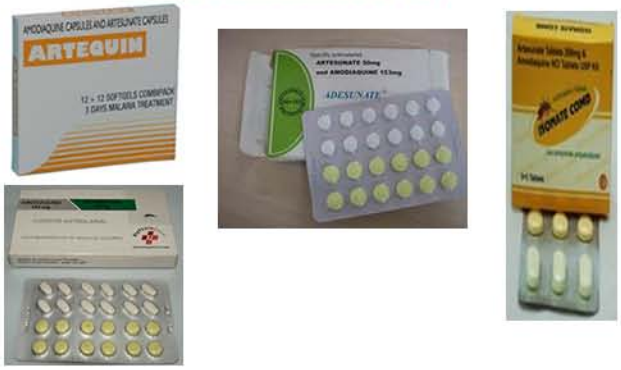

Quinine (M8)

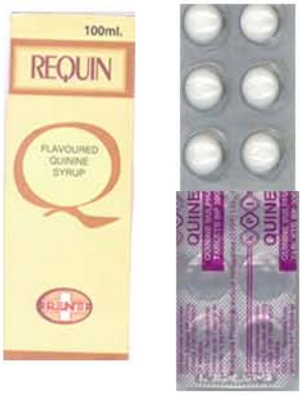

Chloroquine (M9)

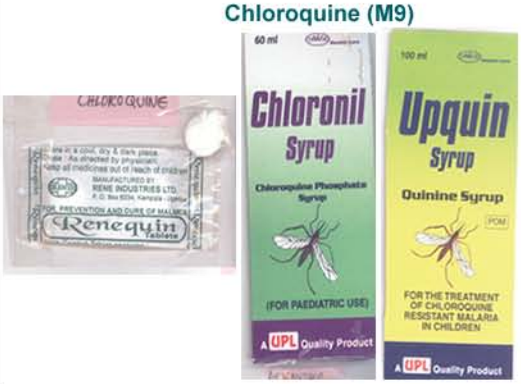

Artesunate Tablets (M10)

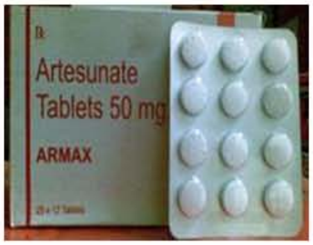

Supplement: S1 Fig — (PDF) [file pone.0200543.s008.pdf]
